# Cr_1/4_NbSe_2_‐Single Crystal Growth and Evolution of Antiferromagnetic Correlated States

**DOI:** 10.1002/advs.78151

**Published:** 2026-10-04

**Authors:** Nour Abdelrahman, Bastian Rubrecht, Matheus Felipe de Souza Barbosa, Mahdi Behnami, Dmitriy Efremov, Daniel Wolf, Martin Valldor, Alexey A. Popov, Stanislav M. Avdoshenko, Marco Naumann, Martin Knupfer, Vladislav Kataev, Alexey Alfonsov, Anja U. B. Wolter, Michael Mertig, Bernd Büchner, Silke Hampel

**Affiliations:** ^1^ Leibniz Institute for Solid State and Materials Research Dresden Dresden Germany; ^2^ Institute of Physical Chemistry Technische Universität Dresden Dresden Germany; ^3^ Helmholtz‐Zentrum Dresden‐Rossendorf e.V. Dresden Germany; ^4^ Department of Chemistry University of Oslo Oslo Norway; ^5^ Kurt‐Schwabe‐Institut für Mess‐ und Sensortechnik Meinsberg e.V. Waldheim Germany; ^6^ Institute of Solid State and Materials Physics Technische Universität Dresden Dresden Germany; ^7^ Würzburg‐Dresden Cluster of Excellence ctd.qmat Technische Universität Dresden Dresden Germany

**Keywords:** antiferromagnetic correlated state, Cr Intercalation, DFT calculations, magnetic anisotropy, reflectivity, resistivity, single crystal synthesis

## Abstract

Transition‐metal dichalcogenides provide a versatile platform for the intercalation of magnetic ions in their van der Waals gaps, enabling tuning of correlated electronic and magnetic states. Despite extensive studies on sulfide‐based systems, Cr*_x_* NbSe_2_ remains poorly understood due to scarcity of single‐crystals and uncertainties in structural analyses leading to contradictory reports on its magnetic behavior. Here, high‐quality single crystals of Cr_1/4_NbSe_2_ via chemical vapor transport were synthesized, along with comprehensive investigation of chemical, structural, electronic, optical, electrical, and magnetic properties, supported by density‐functional theory calculations, presented for the first time. Single crystal x‐ray diffraction, scanning transmission electron microscopy, and selected area electron diffraction reveal ordered Cr intercalation forming a centrosymmetric 2 × 2 superlattice with hexagonal symmetry *P*6_3_/*mmc*. Optical reflectivity indicates an enhanced electron scattering rate. DFT calculations predict antiferromagnetic ground state induced by Cr intercalation. Consistently, SQUID magnetometry and specific heat measurements hint a correlated antiferromagnetic state at *T*_N_ ≈ 107 K, while resistivity measurements show metallic transport, with no signatures of superconductivity. Electron spin resonance suggests weak internal field which possibly arise from subtle magnetic anisotropy, undetectable in angle‐resolved magnetometry. These findings resolve longstanding inconsistencies and establish Cr_1/4_NbSe_2_ as a benchmark vdW antiferromagnet for next‐generation spintronic and quantum materials.

## Graphical abstract

High‐quality single crystals Cr_1/4_NbSe_2_ were synthesized and comprehensively characterized for the first time. Advanced structural, electronic, and magnetic measurements uncover a correlated antiferromagnetic state, resolving longstanding inconsistencies regarding magnetic behavior derived from earlier polycrystal studies. DFT calculations support the experimental observations, establishing Cr_1/4_NbSe_2_ as promising platform for spintronic and quantum magnetic devices.

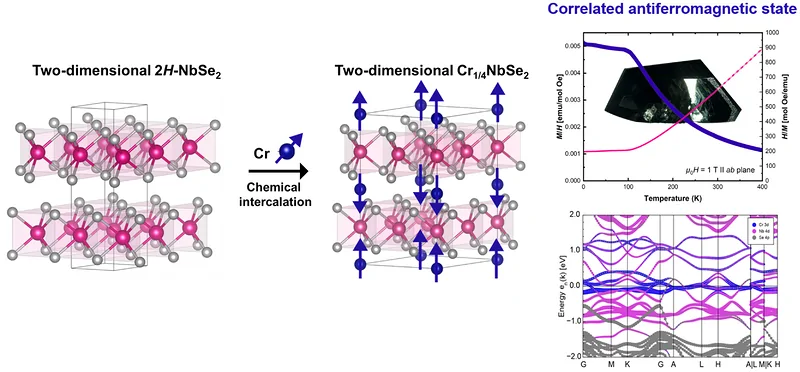

## 1 Introduction

The crystal structures of transition metal dichalcogenides (TMDs) commonly contain two‐dimensional layers that are linked by van der Waals interactions. Such structures allow various species, including transition metals, to be intercalated into the van der Waals gaps in between the layers [1, 2, 3]. Intercalation is a thermodynamically favorable charge transfer reaction, where electron transfer takes place from the intercalant to the host's conduction band, resulting in an electron‐doped lattice with positively charged intercalants [4, 5]. Additionally, intercalants can introduce localized magnetic moments into the host lattice, leading to a variety of magnetic phenomena such as long‐range magnetic order [6, 7], helical spin states [8, 9, 10], noncollinear antiferromagnetic arrangement [11, 12], and anisotropic in‐plane ferromagnetic states [13, 14]. Precisely, These diverse behaviors make intercalated TMDs an ideal platform for exploring the interplay between structure and the resulting optical, electrical, and magnetic properties, thus driving sustained research interest in this class of materials. Intercalation of magnetic atoms such as Fe and Cr in TMDs, e.g. NbS_2_ and TaS_2_, induces a wide range of magnetic behavior that is highly sensitive to both identity and stoichiometry of the intercalant. These intercalated compounds exhibit remarkable functionalities including sharp magnetization switching, [15] low‐current electrical control of antiferromagnetic states [16], and the emergence of chiral spin textures [17, 18], making them promising candidates for next‐generation spintronic technologies. While considerable attention has been devoted to Cr*_x_* NbS_2_, particularly to the well‐studied Cr_1/3_NbS_2_ phase, its selenium analog Cr*_x_* NbSe_2_ remains strikingly underexplored, despite representing a highly promising system with the potential to host similarly rich, composition‐dependent magnetic phenomena. This family of compounds represents another important example of composition‐dependent magnetism. For instance, it has been reported that compositions with 0.05< *x* < 0.33 typically exhibit a cluster‐glass magnetic state, whereas higher Cr contents (*x* ≥  0.33) lead to soft ferromagnetic behavior [7]. Moreover, Cr_1/3_NbSe_2_ is generally characterized as a ferromagnet with a Curie temperature of *T*_c_ ∼ 96–115 K [19, 20, 21], whereas Cr_1/2_NbSe_2_ was suggested to adopt an antiferromagnetic state (*T*_N_ = 53 K, *θ*_CW_ = −141 K) with a ferromagnetic component [22]. Nevertheless, the literature on Cr*_x_* NbSe_2_ remains limited and often contradictory. For example, Cr_1/4_NbSe_2_ has been reported to exhibit a negative paramagnetic Curie‐Weiss temperature (*θ*_CW_ = −40 K) in one study [19], while another one reports a positive value (*θ*_CW_ = +17 K) [23]. These inconsistencies have not been systematically addressed so far and obscure the intrinsic magnetic ground state of this system. Importantly, previous investigations have rarely verified the precise chemical composition of synthesized samples using elemental analysis techniques, such as energy‐dispersive x‐ray spectroscopy (EDX), or inductively coupled plasma optical emission spectroscopy (ICP‐OES). The crystal structure is also rarely described. Furthermore, most magnetic measurements have been performed on polycrystalline powders rather than bulk single crystals [7, 22, 24], leaving important intrinsic properties such as magnetic anisotropy largely unexplored. Comparable difficulties have also been identified in Cr*_x_* NbSe_2_ and related intercalated systems such as Fe*_x_* NbSe_2_, Co*_x_* NbSe_2,_ and Ni*_x_* NbSe_2_, where secondary phases and residual unreacted metallic impurities were detected in polycrystalline powder samples [7, 23]. We argue that these discrepancies primarily originate from variations in sample quality, phase purity, and uncontrolled Cr intercalation amounts, as well as the presence of magnetic impurity phases. Such factors can significantly distort the measured magnetic response and lead to conflicting interpretations. In this work, we address these longstanding inconsistencies by establishing a clear and quantitative relationship between structure, composition, and magnetism in high‐quality single‐crystalline Cr_1/4_NbSe_2_. Through precise compositional control and comprehensive characterization, we provide a unified and consistent interpretation of its magnetic behavior that reconciles previously conflicting reports. We originally explored the synthesis of Cr*_x_* NbSe_2_ compounds with varying chromium concentrations using the chemical vapor transport (CVT) method. CVT is a highly effective technique for producing large, high‐quality bulk crystals, provided that growth parameters are carefully controlled. We obtained the single‐phase material Cr_1/4_NbSe_2_ in very high quality and large dimensions. Comprehensive investigations of these Cr_1/4_NbSe_2_ single crystals were conducted, including structural, electronic, and magnetic characterizations. Detailed analyses of composition, morphology, and crystal structure were complemented by optical studies using Raman spectroscopy and reflectivity measurements. To gain a deeper insight into the electronic properties, density functional theory (DFT) calculations were performed. Furthermore, resistivity measurements were carried out to examine the effect of Cr intercalation on the superconducting state of the pristine 2*H*‐NbSe_2_ lattice. Particular emphasis was placed on elucidating the magnetic properties of Cr_1/4_NbSe_2_, for which SQUID magnetometry, specific heat measurements and electron spin resonance (ESR) experiments were employed.

## 2 Synthesis, Characterization and Crystal Structure

### 2.1 Crystal Morphology and Compositional Analysis

A scheme of the general CVT process of Cr_1/4_NbSe_2_ is illustrated in Figure 1a. The crystal growth occurs in a sealed quartz ampoule placed in a two‐zone furnace with a given temperature gradient. At temperature *T*_1_, the source materials, together with iodine serving as the transport agent, undergo sublimation. The resulting vapor species are then transported toward the lower temperature region at the sink and the condensation on the inner wall of the end side of the ampoule at *T*_2_. After optimizing the growth parameters, the ideal conditions were determined to *T*_1_ = 950°C, *T*_2_ = 800°C, with a temperature gradient ∆*T* = 150 K, maintained for 7 days. Under these conditions, high‐yield black crystals with a metallic luster layered structure were obtained, as depicted in Figure 1c. The crystals exhibited various sizes, predominantly with hexagonal shapes, reaching dimensions up to 6 mm × 3 mm × 0.5 mm (Figure 1c), (see Supporting Information for experimental details).

**FIGURE 1:**
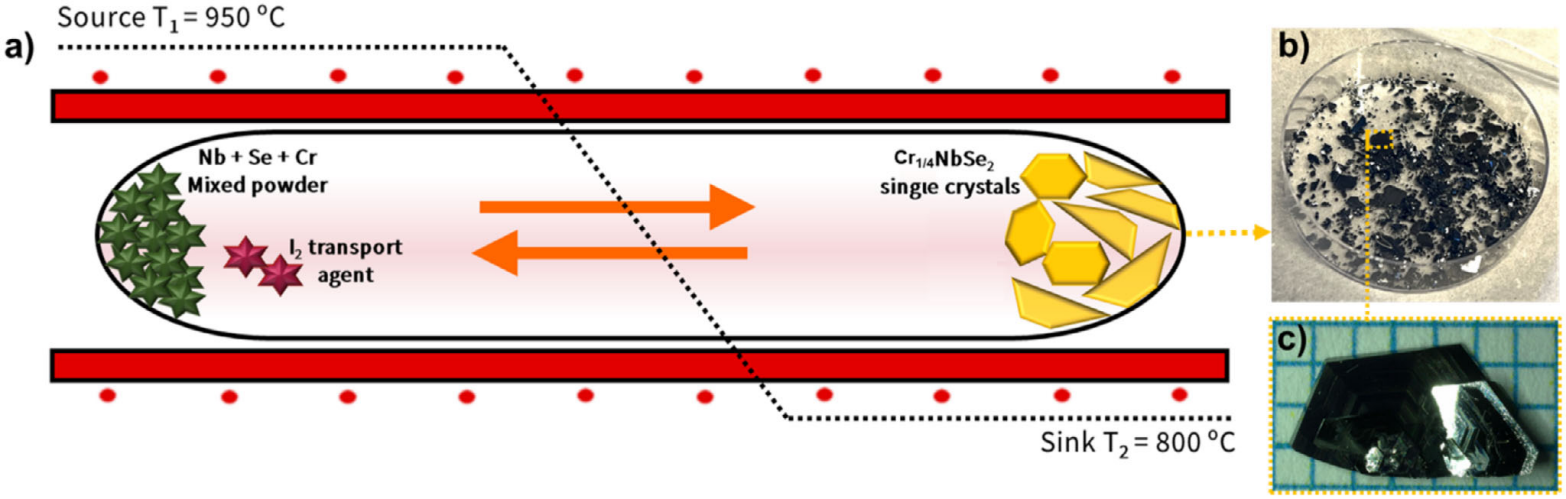
Overview of the growth of Cr_1/4_NbSe_2_ bulk crystals. (a) Schematics of the CVT setup and the synthesis process in a one‐chamber silica ampoule with the starting material placed in the source side and the grown bulk crystals deposited in the sink side. The deposition was enabled by a temperature gradient (see the text for details). (b) High‐yield of Cr_1/4_NbSe_2_ crystals obtained from one CVT experiment. (c) Optical‐microscopy image of a relatively large black shiny crystal with screw‐dislocation.

Well‐defined Cr_1/4_NbSe_2_ crystals exhibiting characteristic edge angles of approximately 120° and a metallic luster were successfully synthesized. The majority of the crystals show prominent screw‐dislocation features, characterized by multiple step edges arranged in a hexagonal symmetry on their surfaces (Figure 2a–d). However, a few crystals with relatively flat surfaces were also observed (Figure 2e). During the growth process, it was common to observe several hexagonally‐shaped crystals clustered together along their edges, indicating a tendency for edge‐to‐edge aggregation (see Figure 2f and additional images, Figure S1).

**FIGURE 2:**
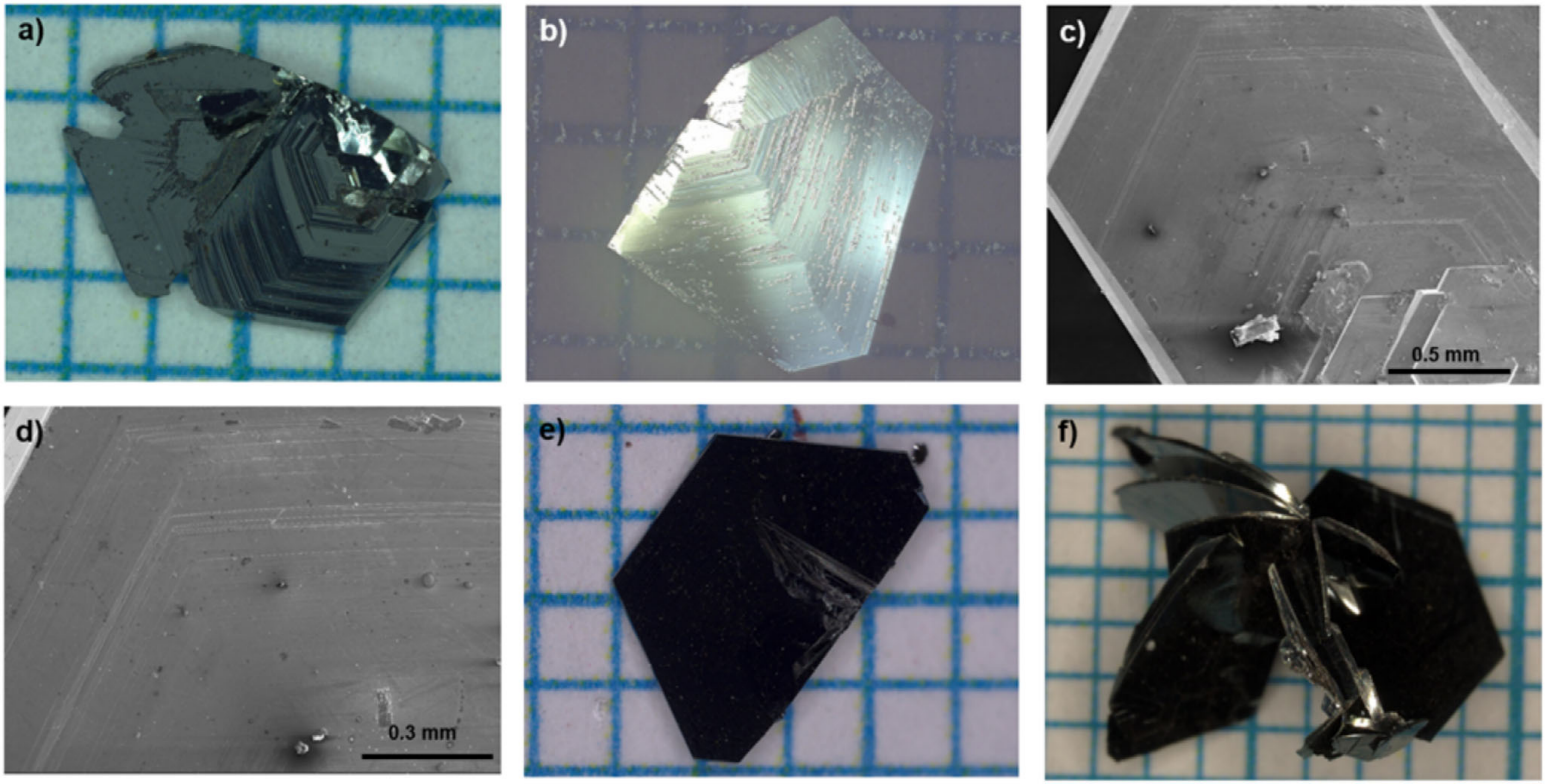
Optical microscopy and SEM images of grown Cr_1/4_NbSe_2_ single crystals exhibiting hexagonal morphology. (a–d) The dominant growth mode observed was screw‐dislocation, resulting in stepped surfaces with hexagonal symmetry. (e) A well‐defined half‐hexagonal crystal with flat surface is shown, apparently iodine residues are visible across the surface, (f) Crystal clustering, where multiple crystals share common edges was frequently observed during the growth process.

One major point in the synthesis is the achievement of the correct composition. The exact elemental composition of the synthesized large single crystals was investigated by inductively coupled plasma optical emission spectroscopy (ICP‐OES). The results, provided in Table S1 show that the nominal chromium value (*x*_nominal_ = 0.30) of the starting material mixture leads to an actual Cr intercalation level (*x* = 0.22) in the obtained crystals after CVT. This is lower than the nominal value of the starting material but obtained for all crystals and can be explained with enrichment effects during the CVT experiments. This observation agrees with several earlier studies on sulfide‐based analog [25, 26]. It also highlights that optimizing *x*_nominal_ is essential for producing Cr_1/4_NbSe_2_ with minimal defects on the Cr sites.

According to EDX analyses, the elemental composition of the crystals matches the expected stoichiometry of Cr_1/4_NbSe_2_ within the intrinsic uncertainty of the technique. Quantitative evaluation based on the averaged results of four area measurements yields an elemental ratio in good agreement with the Cr_1_/_4_NbSe_2_ composition. (Figure S2a). Notably, the EDX measurements were performed on a freshly‐exfoliated surface crystal. Mechanical exfoliation was applied to remove traces of iodine deposited on the surface.

### 2.2 Structural Analysis

In general, the crystal structures of the intercalated system of 2*H*‐Nb*X*_2_ (*X* = S, Se) depend on the intercalation amount of a 3d transition metal, as reported in previous studies [27, 28, 29]. This gives rise to two distinct sets of ordered compounds [29, 30]. When a 3d transition metal element occupies 1/4 of the octahedral holes, it forms an ordered centrosymmetric structure with a 2 × 2 superlattice based on the Nb*X*_2_ unit cell in the hexagonal space group *P*6_3_/*mmc*. In contrast, when the intercalant atom occupies 1/3 of the holes, it creates an ordered √3 × √3 superstructure within non‐centrosymmetric hexagonal space group *P*6_3_22. Cr_1/4_NbSe_2_ falls into the former category, its schematic and crystallographic parameters are depicted in Figure 3 and Table S2. Cr_1/4_NbSe_2_ contains one Cr atom per four NbSe_2_ units, and its unit cell along the *a*,*b*‐axis is twice that of the parent 2*H*‐NbSe_2_ structure. The space group of 2*H*‐NbSe_2_ is retained after intercalation, (see Table S3).

**FIGURE 3:**
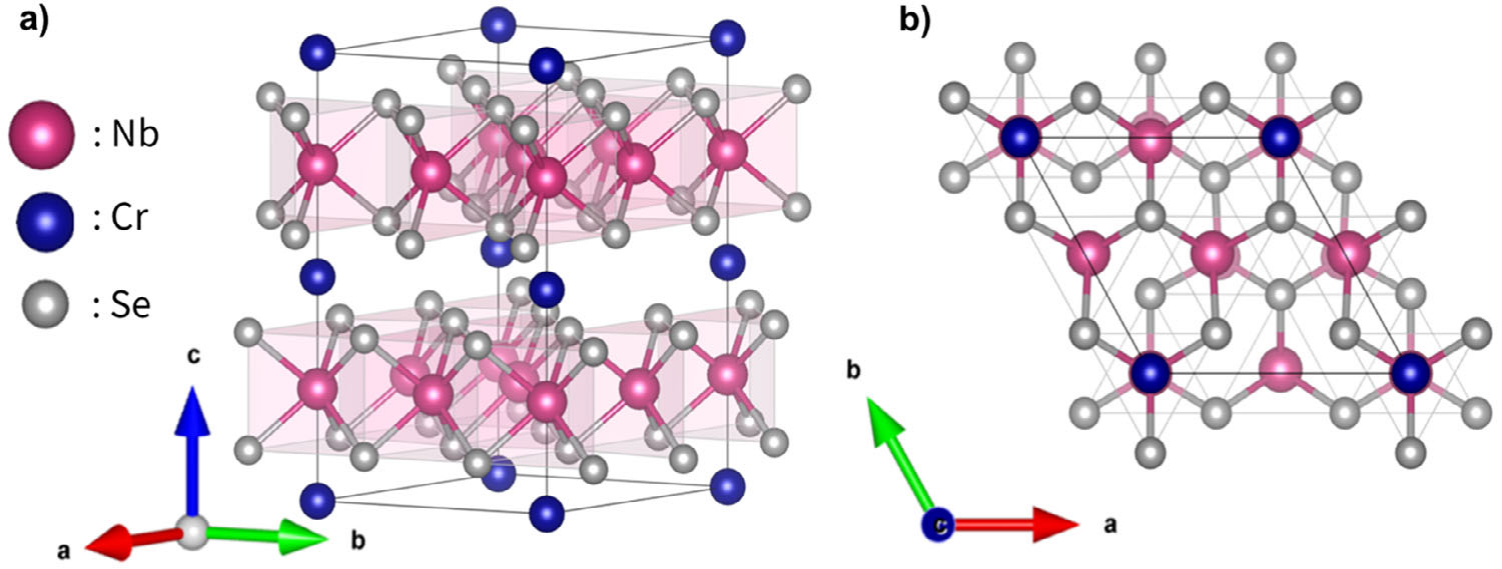
Crystal structure of Cr_1/4_NbSe_2_. Illustrated in side view in (a), while the one along the c‐axis is given in (b). The unit cell of Cr_1/4_NbSe_2_ is given by the solid line.

The Cr–Cr distance of 6.2898 Å indicates that chromium atoms are widely spaced, consistent with their sparse distribution in the intercalated lattice. Cr–Nb distances differ significantly, 4.6715 Å for Cr–Nb1 and 3.1449 Å for Cr–Nb2, reflecting the layered nature of the structure, where Nb atoms in adjacent layers interact differently with intercalated Cr. For Cr–Se, the distances are 4.2296 Å (Cr–Se1) and 2.5084 Å (Cr–Se2). The shorter Cr–Se2 distance suggests a stronger local interaction, likely contributing to structural stability and influencing electronic and magnetic properties. The disparity in Cr–Se distances further highlights the non‐equivalent positions of Se atoms in the crystal lattice. Chromium atoms occupy the 2*a* Wyckoff position, while Nb and Se atoms reside at 6 *h*, 2*b*, 4*f*, and 12*k* sites, respectively, which imparts a centrosymmetric character to the crystal structure. Consequently, the magnetic ordering of Cr_1/4_NbSe_2_ does not exhibit a chiral helimagnetic (CHM) structure as reported in some similar compounds [22, 25, 31], but a correlated antiferromagnetic one as demonstrated in this work. The measured atomic distances and Wykoff positions, obtained using DIAMOND software [32] are presented in Figure S3 and Tables S4 and S5.

The local atomic structure of pristine 2*H–*NbSe_2_ and Cr‐intercalated NbSe_2_ was examined using high‐resolution transmission electron microscopy (HRTEM), scanning transmission electron microscopy in high‐angle annular dark‐field mode (STEM‐HAADF), and selected area electron diffraction (SAED). The results are presented in Figure 4. For pristine 2*H–*NbSe_2_ (Figure 4a–d), the cross‐sectional STEM‐HAADF image (Figure 4a,b), recorded in [100] zone axis orientation, shows a well‐ordered layered structure oriented along the *c*‐axis [001], confirming the typical van der Waals stacking. The atomic columns correspond to Nb and Se atoms arranged in the 2*H* polytype. The Fourier transform of an HRTEM (diffractogram) of the same TEM specimen (Figure 4c), exhibits sharp reflections indexed to the lattice plane fringes (002) and (011), indicating excellent crystallinity and structural uniformity. Consistently, the SAED pattern covers an even larger field of view (Figure 4d). In comparison, Cr_1/4_NbSe_2_ (Figure 4e–h) retains the high crystal quality, but shows distinct differences. The HRTEM image (Figure 4e) is also recorded along [100] zone‐axis orientation, reveals lattice fringes corresponding to planes oriented along [001] direction. In the HRTEM image, the Cr intercalation in between the NbSe_2_ layers is hard to identify due to non‐linear thickness and defocus contrast in HRTEM as even a magnified view (Figure 4f) suggests. However, the Fourier transform of this HRTEM image (Figure 4g) as well as the SAED pattern (Figure 4h) show in addition to one of the NbSe_2_ host lattice reflections (marked by orange arrows), that can be attributed to the superlattice caused by Cr atoms ordering. Overall, these observations demonstrate on the one hand that the pristine NbSe_2_ exhibits perfect 2*H* stacking of the hexagonal *P*6_3_/*mmc* space group with high crystallinity. On the other hand, the Cr intercalation preserves the NbSe_2_ host lattice, but introduces structural changes and superlattice features yielding a 2 × 2 larger unit cell compared to the pristine case.

**FIGURE 4:**
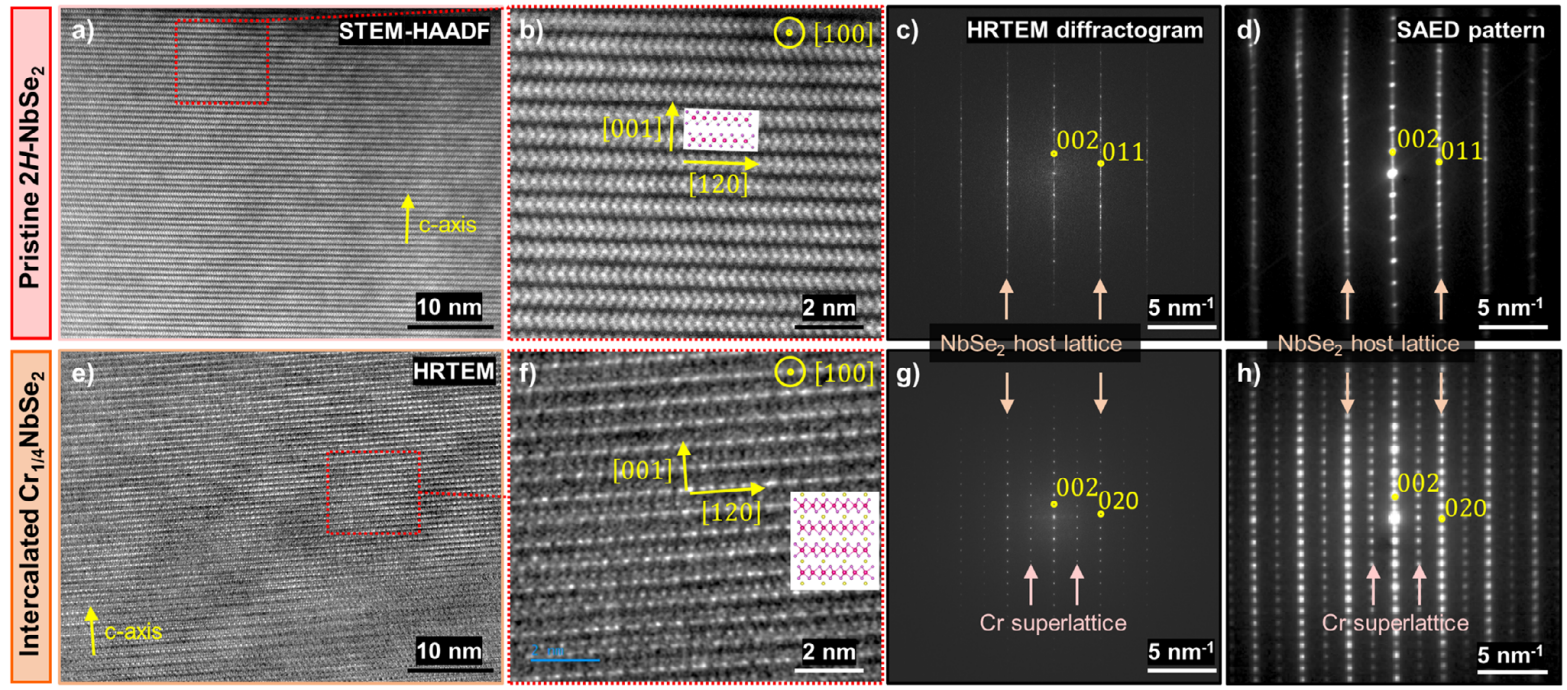
Cross‐sectional TEM of pristine 2H‐NbSe_2_ (a–d), and Cr_1/4_NbSe_2_ (e‐h) single crystals, both recorded along the [100] crystal orientation. (a) STEM‐HAADF image of 2H‐NbSe_2_ crystal near the surface (top). (b) Magnified view of (a), consistent with ICSD 16304 (space group P6_3_/mmc). The inset displays the Nb (dark pink) and, the Se (bright pink). (c) Fourier transform of an HRTEM image. (d) SAED pattern with indexed 002 and* *011 reflections. (e) HRTEM image of Cr_1/4_NbSe_2_. (f) Magnified view of (e). The inset shows the Nb (dark pink), the Se (bright pink), and the Cr (yellow), consistent with (space groupP6_3_/mmc). (g) Fourier transform of an HRTEM image. (h) SAED pattern with 002 and* *020 reflections. Additional reflections (arrows) indicate a Cr superlattice superimposed on the NbSe_2_ host lattice.

## 3 Phonons

Pristine 2*H*‐NbSe_2_ is described by the *D*_6_ *_h_* factor group and has *A*_1_ *_g_* + 2*E*_2_ *_g_* Raman‐active modes in back‐scattering geometry, when both incident and scattered light are propagated perpendicular to the layers. All three modes are straightforwardly observed in experimental spectra excited at 532 nm (Figure 5) or 514 nm [33]. The interlayer shear mode $E_{2}^{1}g$ is detected at 28 cm^−1^. The $E_{2}^{2}g$ mode at 242 cm^−1^ corresponds to lateral displacements of Se and Nb atoms in opposite directions, while *A*_1_ *_g_* at 228 cm^−1^ is the intralayer breathing mode, in which Nb atoms remain still, while Se atoms oscillate perpendicular to the layers. Changing the excitation wavelength to 660 nm enhances intensity of the *A*_1_ *_g_* mode, which now dominates the spectrum, whereas the $\;E_{2}^{2}g$ feature is barely visible. Intensity of the broad two‐phonon peak at 180–190 cm^−1^ and similar features at higher frequencies are also reduced significantly at 660 nm. Similar resonance effects were reported for the excitation at 633 nm [34, 35].

**FIGURE 5:**
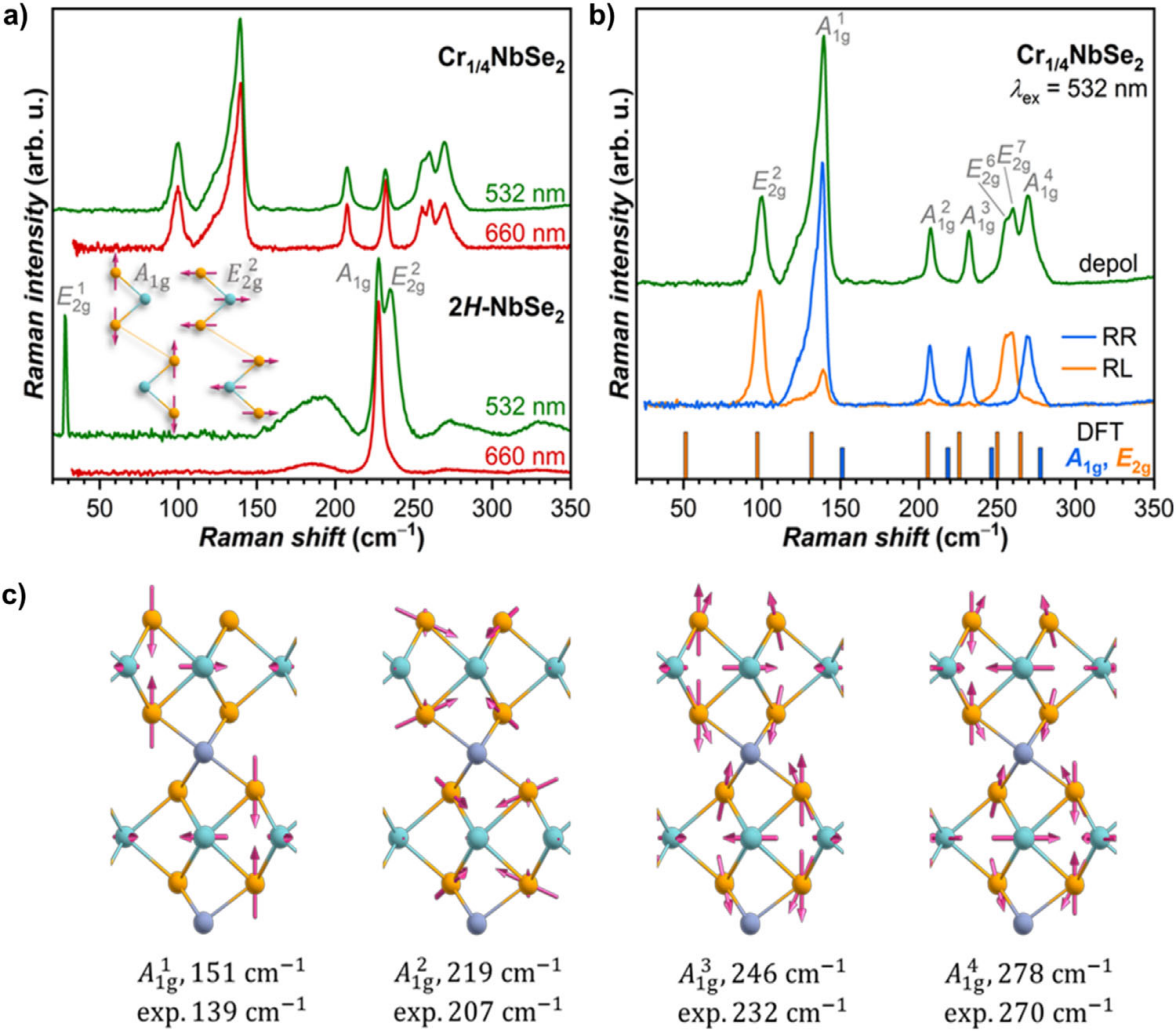
(a) Depolarized Raman spectra of 2H‐NbSe_2_ and Cr_1/4_NbSe_2_ excited at 532 and 660 nm; inset shows vibrational displacement in A_1g_ and ${\;E}_{2}^{2}g$ modes of 2H‐NbSe_2_. (b) Depolarized (“depol”) and circular‐polarized (“RR” and “RL”) Raman spectra of Cr_1/4_NbSe_2_ excited at 532 nm, vertical bars are DFT‐computed vibrational frequencies of Raman‐active A_1g_ and E_2g_ modes of Cr_1/4_NbSe_2_. (c) DFT‐computed atomic displacements for A_1g_ vibrational modes of Cr_1/4_NbSe_2_ along with their computed and experimental frequencies, color code: Nb—cyan, Se—orange, Cr—violet.

In Cr_1/4_NbSe_2_, the factor group symmetry remains the same, but the doubling of the cell parameter increases the number of Raman‐active modes to 4*A*_1_ *_g_* + 7*E*_2g_. Indeed, the intercalation of Cr leads to a considerable enrichment of experimental Raman spectra (Figure 5a). Note that the resonance effect induced by the red laser in NbSe_2_ is suppressed in Cr_1/4_NbSe_2_, and the spectra of the latter excited at 532 and 660 nm are very similar. Interestingly, our Raman spectra of Cr_1/4_NbSe_2_ closely resemble those of Fe_1/4_NbSe_2_ (Table S5) [35, 36]. Measurements with circular polarization were performed to determine symmetry types of the observed modes. For the *D*_6_ *_h_* group, only *A*_1_ *_g_* modes are active when the incident and scattered light have the same chirality (denoted as RR or LL, where R stands for right‐handed and L for left‐handed), whereas only *E*_2_ *_g_* modes are active in the RL or LR settings. This mutual exclusion of *A*_1_ *_g_* and *E*_2_ *_g_* allows facile assignment of all four expected *A*_1_ *_g_* modes in the RR spectrum at 139, 207, 232, and 270 cm^−1^ (Figure 5b). In the RL spectrum, three *E*_2_ *_g_* modes are identified at 100, 256, and 260 cm^−1^. The RL feature at 139 cm^−1^ may be another *E*_2_ *_g_* mode, but its coincidence with the strong $A_{1}^{1}g$ mode may also suggest a polarization leakage. Intensities of three other *E*_2g_ modes are too low to be detected.

To aid in the analysis of the experimental data on the lattice dynamics, DFT calculations of the phonons at the Perdew–Burke–Ernzerhof functional and the Projector augmented wave method (PBE/PAW) level with Vienna ab initio simulation package (VASP) code [36, 37, 38] were performed, see Table S6 for a complete list of phonon modes in the center of the Brillouin zone. Aside from the systematic overestimation of the frequencies by ∼10 cm^−1^, theory gives a good match to all *A*_1_ *_g_* modes. Even a closer agreement is obtained for the three unambiguously assigned *E*_2_ *_g_* modes, suggesting that the missing experimental information can be confidently augmented with computational results. Shear interlayer mode of Cr_1/4_NbSe_2_ is predicted at 52 cm^−1^, whereas the frequencies of three other *E_2g_* modes appear close to those of $A_{1}^{1}g$, $\;A_{1}^{2}g$, and $\;A_{1}^{3}g$.

As the internal structure of NbSe_2_ layers remains almost preserved in Cr_1/4_NbSe_2_, a resemblance of vibrational modes is naturally expected and can be traced by the analysis of the phonon band dispersion and folding in the superlattice. Besides the center‐zone phonons of NbSe_2_, which remain center‐zone phonons in Cr_1/4_NbSe_2_ as well, phonons from the Μ‐point of the original 2*H*‐NbSe_2_ lattice will be also folded to the Γ‐point in the Cr_1/4_NbSe_2_ superlattice [39]. Phonon band structure calculations demonstrate that acoustic and interlayer modes of 2*H*‐NbSe_2_ have relatively strong dispersion and reach 100–130 cm^−1^ in the Μ‐point. Raman peaks of Cr_1/4_NbSe_2_ at 100–150 cm^−1^ can be assigned to such phonons. In analogous transition‐metal intercalated NbSe_2_ compounds they are dubbed as superlattice modes. Dispersion of intralayer phonons of 2*H*‐NbSe_2_ is smaller but still substantial, and in the Μ‐point they span the range of 200–270 cm^−1^, which exactly matches the frequency range of $A_{1}^{2}{g\;-A}_{1}^{4}g$ and $E_{2}^{4}{g-E}_{2}^{7}g$ modes in Cr_1/4_NbSe_2_.

It is tempting to consider that one *A*_1_ *_g_* and two *E*_2_ *_g_* modes of Cr_1/4_NbSe_2_ would correspond to Γ‐point modes of 2*H*‐NbSe_2_, while others originate from the folded Μ‐point phonons. However, as the mixing of the modes with the same symmetry is allowed in the Γ‐point, while *A*_1_ *_g_* and *E*_2_ *_g_* modes can also mix in the Μ‐point [39], preservation of the vibrational forms of 2*H*‐NbSe_2_ in Cr_1/4_NbSe_2_ is not guaranteed. In fact, displacements of *A*_1_ *_g_* modes of Cr_1/4_NbSe_2_ visualized in Figure 5c show that none of them has one‐to‐one match with the *A*_1_ *_g_* mode of 2*H*‐NbSe_2_, and hence it is not possible to designate the latter as a direct predecessor to any of the *A*_1_ *_g_* modes of Cr_1/4_NbSe_2_. Rather, the *A*_1_ *_g_* mode of 2*H*‐NbSe_2_ has a certain contribution to all *A*_1_ *_g_* modes of Cr_1/4_NbSe_2_. Similarly, the $E_{2}^{2}g$ mode of 2*H*‐NbSe_2_ cannot be directly related to one specific Cr_1/4_NbSe_2_ phonon. Only the shear ${\;E}_{2}^{1}g$ mode has a close resemblance with the ${\;E}_{2}^{1}g$ mode of the intercalated compound. Its shift to the higher frequency in Cr_1/4_NbSe_2_ is presumably induced by the increase of the curvature of the potential energy surface for the sliding of NbSe_2_ layers along each other caused by the Cr─Se bonding. Additional details on the DFT‐computed Γ‐point vibrational modes, including vibrational displacements of *A*_1g_ and *E*_2g_ modes of Cr_1/4_NbSe_2_, are provided in Figures S4, S5 and Tables S6 and S7, and in the Supporting Information.

## 4 Electronic and Optical Properties

### 4.1 Density‐Functional‐Theory (DFT) Calculations

DFT calculations were performed using the full‐potential local‐orbital band structure (FPLO) package [40, 41]. Exchange and correlation were treated within the generalized gradient approximation (GGA/PBE); no on‐site Hubbard U was applied to the Cr 3d states [42]. For the non‐spin‐polarized calculations, the experimental crystal structure of Cr_1/4_NbSe_2_ presented in this work was used as the input structure, whereas a 2 × 2 × 1 supercell was employed for the spin‐polarized calculations. Magnetic Brillouin‐zone integrations were carried out using a 6 × 6 × 3 k‐point mesh. The lattice parameters and space‐group symmetry were kept fixed to the experimental values.

Figure 6 compares the electronic band structure of pristine 2*H*‐NbSe_2_ and Cr_1/4_NbSe_2_ in the paramagnetic state using the orbital‐projected density of states (DOS) and orbital‐resolves band structures (Note that the unit cell of the of pristine 2*H*‐NbSe_2_ is four times smaller than Cr_1/4_NbSe_2_). In 2*H*‐NbSe_2_ (Figure 6b,d), the electronic states in the vicinity of the Fermi level and above are primarily derived from Nb 4𝑑 orbitals, while below the Fermi energy the dominating contribution is from Se 4p. The band structure is consistent with the metallic character of layered transition‐metal dichalcogenides. In Cr_1/4_NbSe_2_ (Figure 6a,c), the DOS acquires a clear Cr‐derived component. The DOS indicates that the 3d states of Cr contribute considerably near the Fermi energy within the energy window of—6 to 5 eV, in both the non‐polarized and polarized spin cases. In the spin‐polarized calculations, the 3d electron spectra of Cr are strongly spin polarized, with pronounced exchange splitting between the two spin channels (see Figures S6–S9). As the 3d‐orbitals of Cr are itinerant, the projection onto the local orbitals may differ depending on the projection method. The FPLO population analysis suggests that the effective spin is approximately 3/2 for the Cr atoms, which mostly originate from the 3d Cr orbitals. The occupation numbers of the 3d Cr orbitals are approximately 4.3 and 4.8 net and gross correspondingly.

**FIGURE 6:**
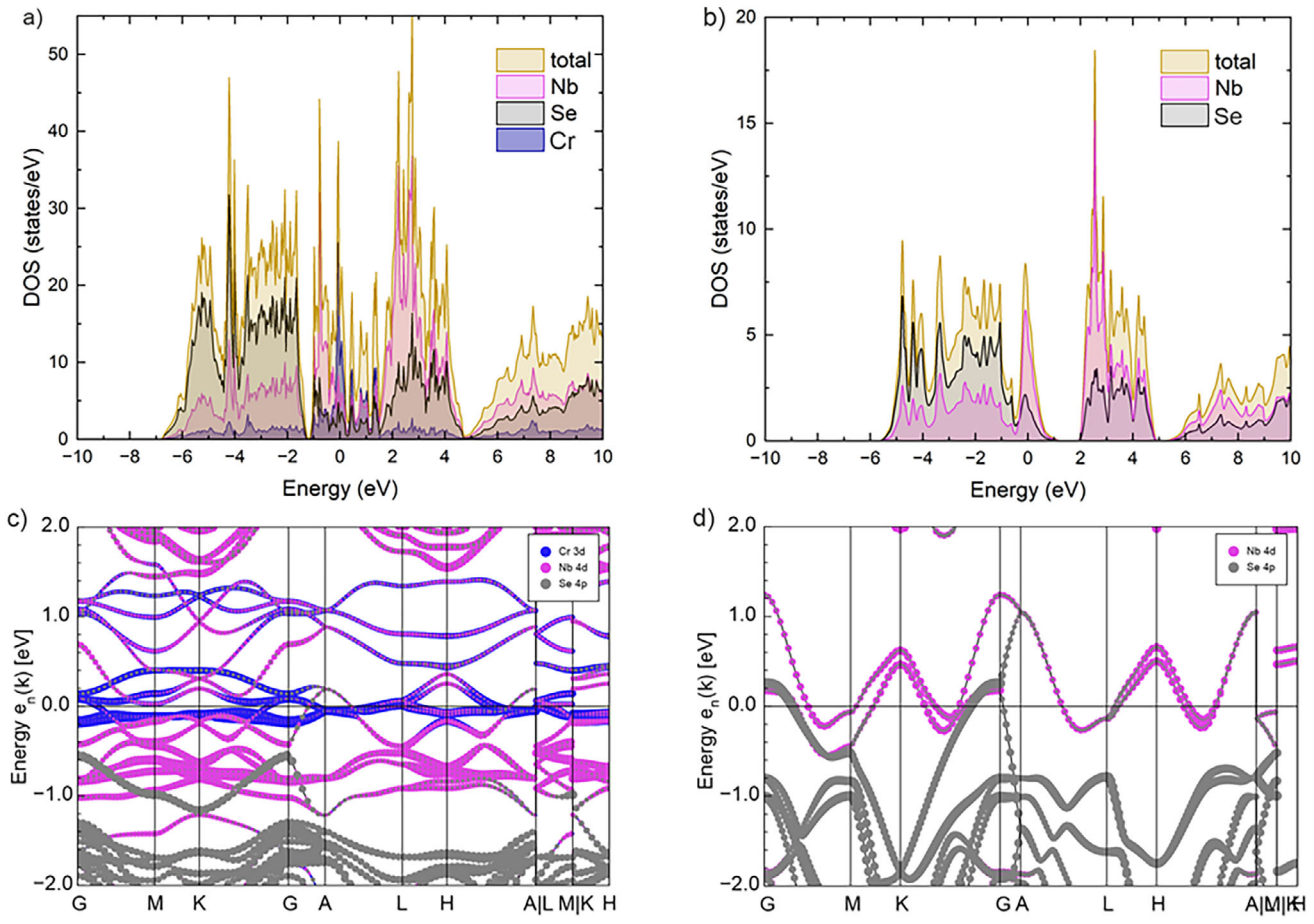
Atomically projected (element‐resolved) non‐spin‐polarized density of states (DOS) of: (a) Cr_1/4_NbSe_2_ and, (b) NbSe_2_. Atomically and orbital projected, non‐spin‐polarized electronic bands of (c) Cr_1/4_NbSe_2_, and (d) NbSe_2_. Line widths are proportional to the contributions of the corresponding atomic orbitals. Note that the Cr_1/4_NbSe_2_ unit cell is four times larger than that of NbSe_2_.

To quantify the magnetic interactions, we computed spin‐polarized total energies (per Cr) for a ferromagnetic (FM) configuration and two antiferromagnetic (AFM) configurations: (i) A‐type AFM, consisting of ferromagnetic layers stacked antiferromagnetically along the 𝑐 direction, and (ii) an in‐plane stripe AFM order with ferromagnetic stripes coupled antiferromagnetically.

The energy differences were mapped onto a nearest‐neighbor Heisenberg Hamiltonian with spin *S* =  3/2:

$$H=1/2\sum_{<ij>}JS_{i}S_{j}+1/2\sum_{<ij>}J_{c}S_{i}S_{j}$$

Yielding an antiferromagnetic intraplane coupling *J*  =  1.5 *meV*, and ferromagnetic interplane coupling *J_c_* =  ‐15 *meV*,  the values consistent with the exchange constants obtained earlier in ref [30]. The positive Curie‐Weiss temperature *θ*_CW_ = +51 K is dominated by the large ferromagnetic interplane coupling, whereas the antiferromagnetic ground state originates from the weaker, frustrated antiferromagnetic coupling within the triangular Cr planes; a positive *θ*_CW_ is thus fully compatible with 120° antiferromagnetic order subsequently reported from neutron diffraction on Cr‐intercalated NbSe_2_ [43]. We note that in a metallic system the Curie‐Weiss analysis is only phenomenological and does not directly yield the microscopic exchange constants.

### 4.2 Reflectivity Measurements

In Figure 7 the measured reflectivity of an as‐grown Cr_1/4_NbSe_2_ crystals between 50 meV and 1.3 eV is depicted. The data have been measured in normal incidence geometry on the *ab*‐plane of the crystal. Moreover, the measured reflectivity data have been fitted using a simple Drude model, where the dielectric function is given by [44]:

$$\varepsilon \;\left(\omega \right)=\varepsilon_{\infty }\;-\;\frac{\omega_{p}^{2}}{\omega^{2}+i\omega \Gamma }$$

**FIGURE 7:**
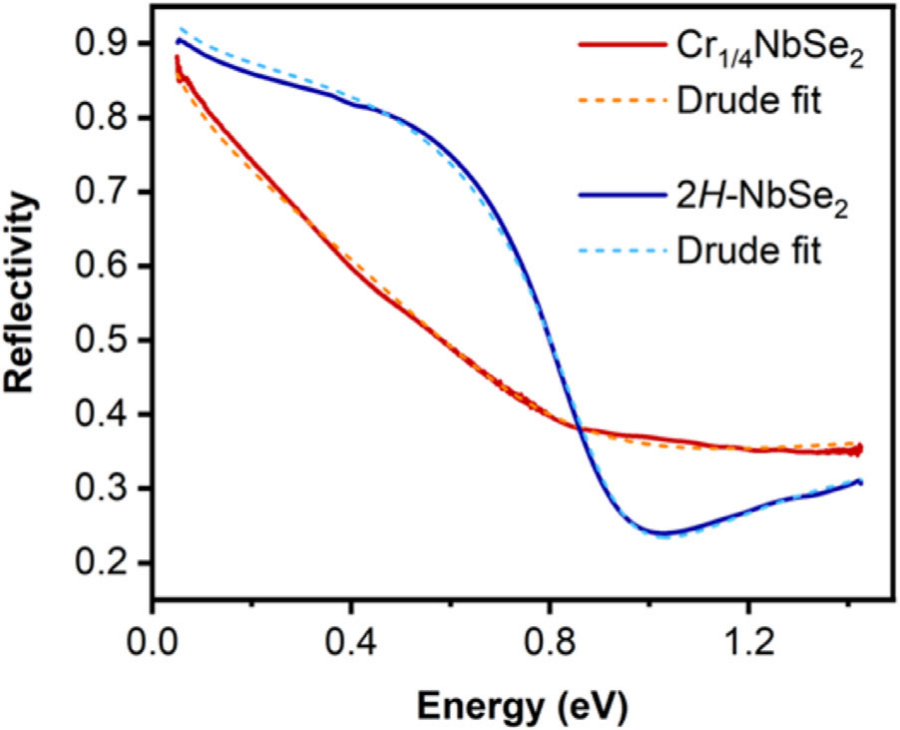
Reflectivity data of Cr_1/4_NbSe_2_ crystal (red line) and a 2H‐NbSe_2_ single crystal (blue line) measured in normal incidence geometry at the ab‐plane, respectively. The corresponding Drude fits are shown as dashed lines, (see text for details).

Here ε_∞_ denotes the background dielectric constant due to higher energy inter‐band excitations, ω_*p*_ =  (*ne* ^2^/ε_0_ m*)^1/2^ is the plasma frequency, Γ represents the scattering rate of the conduction electrons, *n* is the conduction electron density, e the elementary charge, *m* ^*^ the (effective) mass of the conduction electrons, and ε_0_ the vacuum permittivity. The result of this fitting is shown in Figure 7. For comparison, the reflectivity data of a 2*H*‐NbSe_2_ single crystal with the corresponding Drude fit were additionally included. The results of data fitting are summarized in Table 1.

**TABLE 1:** Results of a Drude fit of the reflectivity data of 2H‐NbSe_2_ and Cr_1/4_NbSe_2_ crystals.

Material	***ε***_∞_	***ω***_***p***_ * *(***eV***)	***Γ*** (eV)
2H‐NbSe_2_	20.5	4.19	0.31
Cr_1/4_NbSe_2_	20.9	3.94	0.9

The analysis demonstrates that the simple Drude approach as chosen here can well reproduce the measured reflectivity data for both materials. 2*H*‐NbSe_2_ is a well‐known 2D metal (see also Figure 6 above) and it has been widely investigated due to competing charge‐density‐wave (CDW) and superconducting ground states [45]. Its reflectivity as measured in this work agrees well with previously published data [46, 47, 48]. It is characterized by a sharp plasma edge at about 0.8 eV, and the following minimum reflects a quite large contribution of energetically higher lying inter‐band excitations to the reflectivity. This is represented by the background dielectric constant *ε_∞_* ∼20, see Table 1. Going to chromium intercalated Cr_1/4_NbSe_2_, the reflectivity changes significantly. Our data show a gradual decrease of the reflectivity over a rather large energy range instead a sharp plasma edge. This signals a significant increase of the conduction electron scattering rate as compared to 2*H*‐NbSe_2_. Indeed, our Drude fit reveals a scattering rate being about a factor of 3 larger in Cr_1/4_NbSe_2_, while the plasma frequency and the background dielectric constant only slightly change. Band structure calculations (see Figure 6) reveal the presence of hybridized flat bands with substantial Cr character close to the Fermi level. Consequently, we expect low‐energy inter‐band excitations related to these bands that will enhance the charge carrier scattering rate. Moreover, the larger scattering rate also may arise from an increased scattering with Cr‐related phonons.

## 5 Electrical Properties

### 5.1 Resistivity and Magneto‐Transport Measurements

The temperature‐dependent resistivity of Cr_1/4_NbSe_2_ and 2*H*‐NbSe_2_ single crystals is presented in Figure 8a, spanning from room temperature down to 1.8 K. Both samples exhibit metallic behavior across the measured temperature range. However, it is clearly demonstrated that Cr intercalation completely suppresses the transition into superconducting state, which is observed for pristine 2*H*‐NbSe_2_ at *T*_c_ = 6.7 K. This behavior is expected when magnetic elements such as Cr are intercalated into superconducting materials, as their magnetic moments disrupt Cooper pairing. Resistivity and magneto‐transport measurements for pristine 2*H*‐NbSe_2_ are shown in Figure S10. A distinct change in the resistivity slope is observed, accompanied by two sharp anomalies near 94 and 108 K in the derivative plot (Figure 8b). These anomalies are likely associated with magnetic ordering, as evidenced by magnetic measurements discussed later. The observed decrease in resistivity below the Néel temperature suggests reduced spin fluctuations, a hall mark of magnetic ordering [49]. Figure 8c shows the magneto‐resistance (MR) as a function of magnetic field applied perpendicular to the sample plane, at various temperatures. The observed MR is negative across the entire temperature range, varying from approximately −0.1% to −0.45%, which is characteristic for magnetically ordered systems, where the applied field suppresses spin fluctuations and consequently lowers the resistivity [50]. At low temperatures, magnetic scattering dominates; thus, applying a field below the Néel temperature significantly reduces spin fluctuations, resulting in a pronounced negative MR. At higher temperatures, phonon and impurity scattering become more prominent, diminishing the magnetic field's influence and reducing the magnitude of negative MR.

**FIGURE 8:**
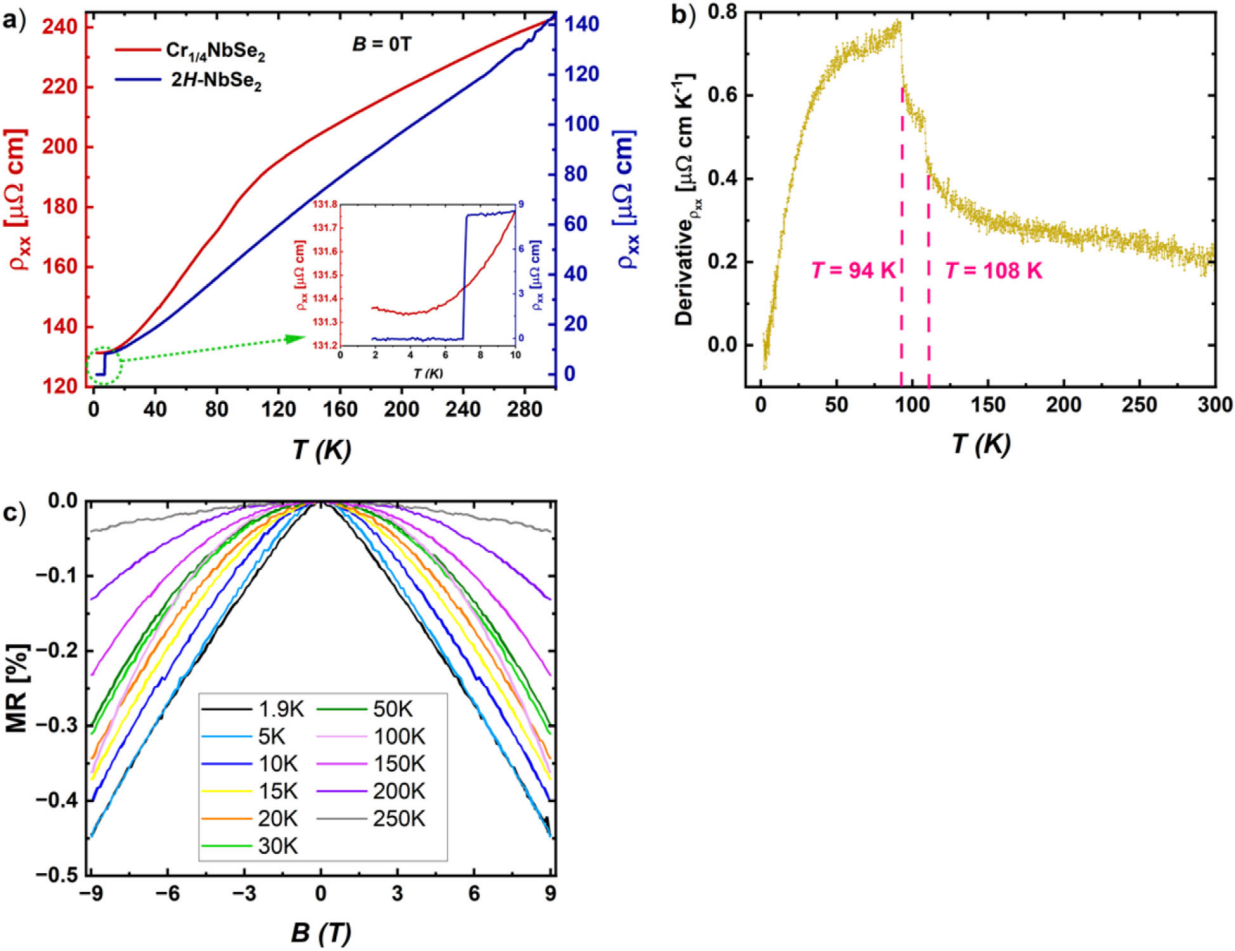
(a) Comparison of temperature‐dependent resistivity between Cr_1/4_NbSe_2_ and pristine 2H‐NbSe_2_ single crystals. The inset shows a zoom onto the low‐temperature‐dependent resistivity from 2 to 10 K. (b) Temperature derivative of the resistivity. (c) Magnetoresistance of Cr_1/4_NbSe_2_ single crystals as a function of magnetic field at different temperatures.

## 6 Evolution of Magnetic Properties

### 6.1 Bulk Magnetometry and Specific Heat Measurements

Figure 9a shows the magnetization divided by the applied field (*M/H*) as well as it's inverse (*H/M*) as a function of temperature for *H II ab*‐plane. At high temperature, the magnetic susceptibility follows a paramagnetic Curie‐Weiss law. By restricting the analysis to T > 300 K and fitting the inverse susceptibility, [*H*/*M* = (*C* / (*T*—*θ*_CW_) + *χ_0_*) ^−1^], we obtain an effective Cr moment of µ_eff_ = (3.6 ± 0.1) µ_B_/Cr, a Curie‐Weiss temperature of *θ*_CW_ = (51 ± 10) K and a temperature independent contribution of *χ*_0_ = −(9 ± 2)10^−5^ emu/mol Oe, assuming the ideal composition Cr_1/4_NbSe_2_. The moment closely matches the spin‐only value of 3.87 μ_B_ corresponding to *S* = 3/2 configuration with a quenched orbital momentum. However, the absence of the single ion anisotropy suggests, that the electronic configuration of Cr is different from a canonical Cr^3+^ ion. Cr‐Nb hybridization leads to a redistribution of electronic charge between Cr and the NbSe_2_ layers. In particular, this scenario was argued to be realized for Cr_1/3_NbSe_2_ and Cr_1/2_NbSe_2_, where NMR data indicate a finite spin polarization at the Nb atoms [7]. The positive Curie–Weiss temperature obtained from our fit for Cr_1/4_NbSe_2_ hints at ferromagnetic correlations (in line with the ferromagnetic order along the c direction predicted by our DFT calculations), although in a metallic system the Curie‐Weiss formalism is only phenomenological and does not directly reflect microscopic exchange constants. Below *T*_N_ = 107 K the magnetic susceptibility no longer displays a monotonic increase; instead, its temperature dependence changes abruptly resulting in two broad kinks followed by a rather constant susceptibility at low temperature. The position of the two kinks in *M*/*H* does not change up to the maximum applied field of 7 T, and their modest amplitude suggests a gradual onset of antiferromagnetic correlations, in line with our specific heat measurements (see below). The field dependence of the magnetization at 1.8 K is shown in Figure 9b. At *T* = 1.8 K the magnetization *M (H)* is strikingly linear across the full field regime, reaching ± 0.062 µB/f.u. (or ∼ ±0.25 µB/Cr) at ± 7 T, again supporting an antiferromagnetic correlated state. Only in the immediate vicinity of zero field the sample shows a small hysteresis loop, whose most plausible origin is a very small Cr‐based secondary phase rather than an intrinsic property of the host lattice. Based on the magnetization data, we estimate the saturation moment of the secondary phase to be around 0.6 × 10^−^^3^ to 3.4 × 10^−^^3^ µB/Cr. This translates to an upper limit of 0.2 at% Cr and a lower limit of 0.01 at% Cr for the secondary‐phase fraction, already accounting for different possible oxidation states of the Cr ions. Figure 9c shows the angular dependence of the magnetic moment from the *ab* plane towards the c direction at various fields and temperatures. The signal is essentially isotropic; a very small variation of the amplitude of < 10% probably originates from the angular dependence of the rotator background, given the small raw moment of the sample (∼7 × 10^−5^ emu at 50 K, 0.1 T), and is also present above the Néel temperature. Finally, the specific heat of Cr_1/4_NbSe_2_ was measured in zero magnetic field between 1.8 and 350 K (Figure 9d). *C*_p_ increases smoothly with temperature and approaches the Delong‐Petit limit at high temperatures, without exhibiting a sharp thermodynamic phase transition. After post‐processing [51], however, a very weak and rather broad anomaly becomes discernible as a subtle kink around 107 K (see inset). As specific heat is a bulk‐sensitive probe, this kink is consistent with the onset of antiferromagnetic correlations throughout the sample volume. While the broad While the broad anomaly does not constitute direct evidence for long‐range order, it is fully compatible with such an interpretation. Similar broadened thermodynamic signatures are commonly observed in frustrated layered AFM [52], where strong short‐range correlations and spin fluctuations persist well above *T*_N_. Such an additional broad magnetic contribution, however, is likely masked by the dominant phonon background in our case.

**FIGURE 9:**
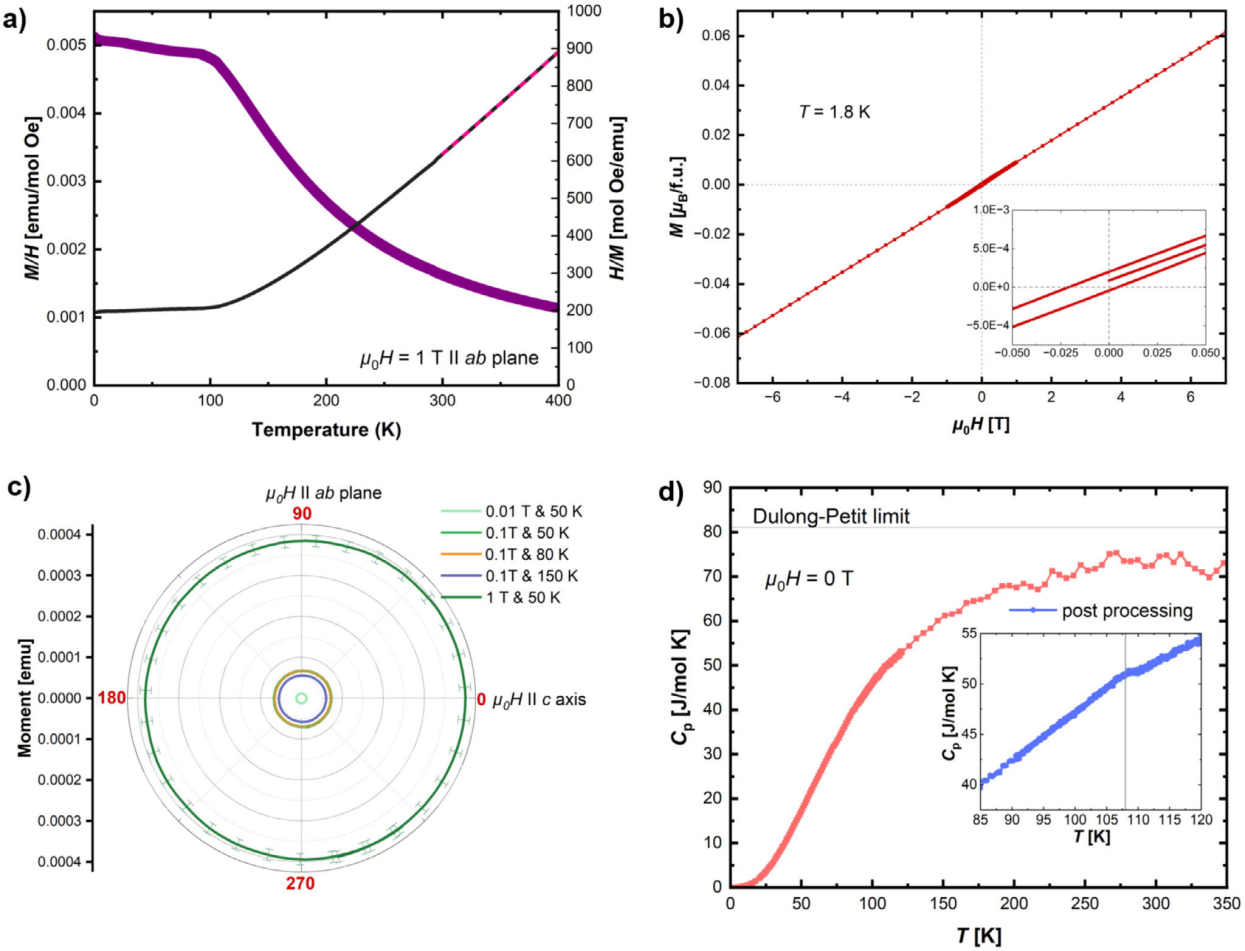
(a) Temperature dependence of the magnetic moment divided by the applied magnetic field and its inverse. The applied field of 1T is parallel to the crystallographic ab plane. (b) Magnetization hysteresis loop M(H) measured at 1.8 K. The insert shows a zoom onto the low‐field regime from −300 to + 300 Oe. (c) Angular dependence of the magnetization M(θ) displayed in polar coordinates for various fields and temperatures. Here, 0° and 90° correspond to H parallel ab plane and H parallel to c axis, respectively. (d) Temperature dependence of the specific heat C_P_(T) at zero field. The insert shows a comparison of the as measured vs. post‐processed data around the transition temperature of 107 K.

Overall, our findings differ from earlier reports on Cr_1/4_NbSe_2_: Toporova et al. [7] reported a spin‐/cluster glass transition at *T*_C_ = 52 K (see Figure S11) and van den Berg [22] observed ferromagnetism below *T*_C_ = 79 K. Both studies reported a pronounced ZFC‐FC splitting at low fields below *T*_C_, a feature absent in our data down to 100 Oe. The temperature dependence of *M/H* and the linear field dependence *M (H)* determine a correlated antiferromagnet state, while our angular measurements show virtually no magnetic anisotropy. Note that after submission of this manuscript, Yamaoka et al. [43] reported neutron‐scattering evidence for a long‐range 120° AFM order in Cr‐intercalated NbSe_2_. While their crystals possess a significantly reduced Cr occupancy (*x* ∼ 0.186), likely accounting for the lower ordering temperature, their magnetization data exhibit the same broad anomaly and plateau‐like behavior as observed in our measurements. Combined with the absence of frequency dependence in our AC susceptibility, excluding a glassy magnetic state (Figure S11), these findings support the interpretation that our Cr_1/4_NbSe_2_ samples could realize the same long‐range AFM ground state. However, future neutron diffraction experiments are needed to unambiguously determine the microscopic spin structure in our stoichiometric samples with x = 0.25.

### 6.2 Electron Spin Resonance Measurements

To further investigate the magnetic properties of the high‐quality single crystal of Cr_1/4_NbSe_2_, we performed high‐field/high‐frequency electron spin resonance (HF‐ESR) measurements at various microwave frequencies, temperatures and at two orientations of the magnetic field with respect to the crystal (*H* ∥ *ab*) and (*H* ⊥ *ab*). Figure 10a shows the temperature dependence of the spectra measured at a frequency of ∼75 GHz. At elevated temperatures the ESR signal from Cr_1/4_NbSe_2_ is not observed. The signal emerges at around 150 K and further develops by reducing the temperature. Although the ESR response of the sample is weak and its shape is complex, a weak temperature dependence of the resonance line is evident. For both magnetic field orientations, in measurements performed at the frequency of ∼75 GHz, a complex‐peak structure is observed at low temperatures. Interestingly, there is an observable shift of the low field shoulder towards the paramagnetic position when the temperature approaches ∼100 K from below (Figure 10a). This characteristic temperature is consistent with the transition temperature to antiferromagnetic correlated state (∼107 K) obtained by bulk magnetometry and specific heat measurements (see above). In order to estimate the effective g‐factor and to possibly find the signatures of magnetic anisotropy in the ordered state, frequency dependent measurements at *T* = 3 K was performed (Figure 10b,c)). At different excitation frequencies the spectra show complex lines shapes, in line with the temperature dependence measurements. Due to such a complex shape, it was not possible to reliably fit the ESR response with Lorentzian line profile. Therefore, to estimate the effective resonance field *H*_res_ of the whole structure of the overlapping resonance lines we use its midpoint, and the error bars are taken as its estimated total width. The fit of this data using linear dependence of the microwave frequency on the resonance field (*hν* = g µ_B_µ_0_ *H*_res_ +*∆*) provides an estimate of the g‐factors *g*_∥_ = (1.97 ± 0.03) and *g*_⊥_ = (2.02 ± 0.02), corresponding to the in‐plane (*H* ∥ *ab*) and out‐of‐plane (*H* ⊥ *ab*) orientations, respectively. These g‐factors are very close to the g‐factors expected for Cr ion with orbital angular momentum quenched to first order [53]. The zero‐field excitation gap, which in the first approximation is given by the intercept *∆*, is found to be zero within the error bars. Although the g‐factors extracted for both field orientations, *g*_∥_ and *g*_⊥_, are similar within experimental uncertainty and the zero field gaps are close to zero, the temperature‐dependent shift of the resonance lines toward lower magnetic fields at around ordering temperature suggests a possible presence of an effective internal field whose origin could be in the weak magnetic anisotropy of the system. This anisotropic effect seems to be subtle, which may explain why it is not detected in angle‐dependent SQUID measurements (Figure 9c), and is also hidden behind a complex shape of the ESR line.

**FIGURE 10:**
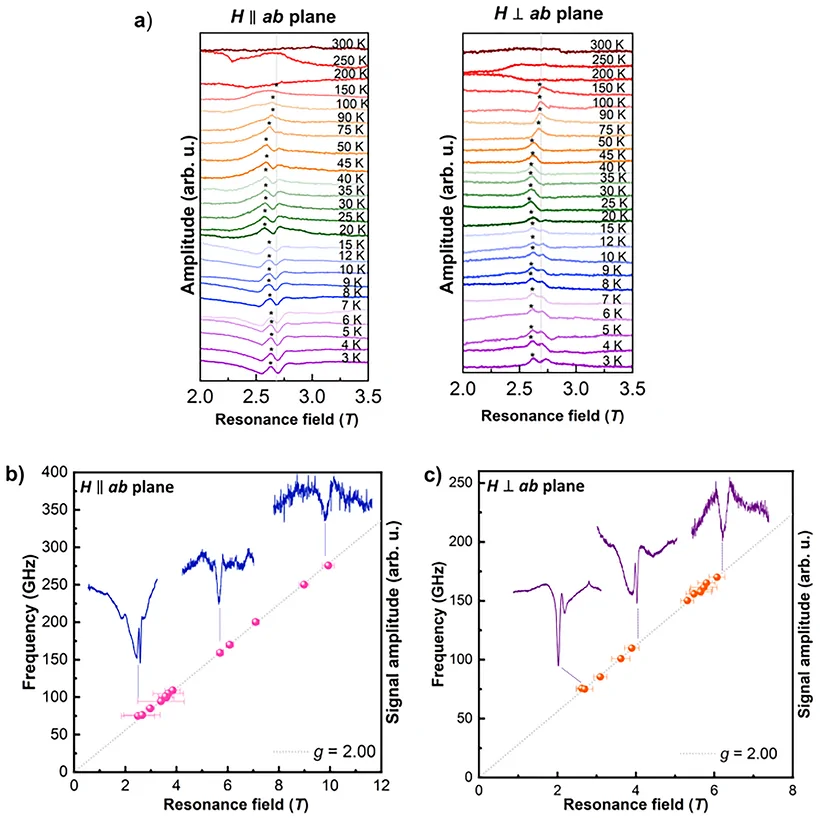
High‐field ESR experiments performed on Cr_1/4_NbSe_2_. (a) Temperature dependence of the ESR signal for *H* ∥ ab‐plane (left, frequency 75.0327 GHz) and *H* ⊥ *ab*‐plane (right, frequency 75.3558 GHz). The vertical dashed line indicates the paramagnetic resonance line at the field corresponding to g = 2 and (*) mark is a guide for the eyes, representing the temperature evolution of the peak position. (b, c) Left vertical scale: Frequency dependence of the resonance field at 3 K for applied magnetic field in‐plane (b) and out of plane (c), error bars are taken as estimated width of the spectra. Dashed lines represent the paramagnetic response with the g‐factor equal to 2. The linear fit of the experimental data (see text for details) provides an estimate of the g‐factors *g*_∥_ = (1.97 ± 0.03) and *g*_⊥_ = (2.02 ± 0.02), representing in‐plane and out‐of‐plane configurations, respectively. Right vertical scale: Exemplary ESR spectra at selected frequencies.

## 7 Conclusion

In this work, we have established Cr_1/4_NbSe_2_ as a well‐defined and experimentally robust model system for studying magnetic intercalation in layered transition‐metal dichalcogenides. High‐quality single crystals with precisely controlled and verified Cr stoichiometry were successfully synthesized for the first time, overcoming long‐standing ambiguities in composition and phase purity that have limited previous investigations. Sc‐XRD, STEM confirm an ordered Cr intercalation within a centrosymmetric 2 × 2 superlattice. Optical reflectivity reveals significant reconstruction of the electronic band structure near the Fermi level. This finding is consistent with our DFT calculations, which show a strong contribution of 3d electrons to the band structure near the Fermi level. Further, spin‐resolved calculations reveal antiferromagnetic Cr–Cr exchange interactions within the planes and ferromagnetic interactions between the planes, which may stabilize antiferromagnetic ground states, including noncollinear configurations. Indeed, SQUID magnetometry and specific heat indicated the emergence of a correlated antiferromagnetic state below *T*_N_ ≈ 107 K. In agreement with this, resistivity measurements reveal a clear change in slope together with a pronounced anomaly in the temperature derivative at *T*_N_, providing further evidence for the antiferromagnetic transition. No signatures of superconductivity were detected down to the lowest temperatures accessible in the experiment. ESR suggests a possible presence of an effective internal field whose origin could be in the weak magnetic anisotropy of the system. By resolving previous inconsistencies through precise compositional control and comprehensive characterization, our results provide a unified and physically consistent description of the magnetic ground state in Cr_1/4_NbSe_2_. This work represents the first comprehensive and internally consistent study of this system, and further motivate systematic investigations of the influence of variations in intercalant stoichiometry, dimensionality reduction, and synthesis conditions on magnetic properties in selenide‑based intercalated systems. Such systems remain comparatively underexplored relative to sulfide analog and offer promising opportunities for controlled magnetic engineering in layered materials.

## 8 Experimental Section

### 8.1 Synthesis of Pristine NbSe_2_ and Intercalated Cr_1/4_NbSe_2_ Bulk Crystals

All preparation steps were performed in a glovebox (M. Braun). The ampoules used for CVT growth were one‐chamber ampoules with dimensions of 16 cm in length, 1 cm inner diameter, 0.5 mm neck diameter, and a volume of approximately 20 mL. They were fabricated from fused silica (ilmasil PN, qsil, max. 45 ppm OH content). The ampoules were evacuated and sealed at a pressure of ∼2 × 10^−3^ mbar). Pristine NbSe_2_ single crystals were grown in one‐step chemical vapor transport by loading 1 g of the stoichiometric amounts of niobium powder (> 99.99%, Thermo Scientific), and selenium powder (> 99.99%, Alfa Aesar) together with 0.08 g of iodine (double sublimed, analytical grade, Merck) into an evacuated silica ampoule. The sealed ampoule was placed in a horizontal two‐zone tube furnace (LOBA, HTM Reetz GmbH), with the hot zone maintained at 950°C and the cold zone at 800°C for 7 days. After natural cooling to room temperature, NbSe_2_ single crystals were obtained. Subsequently, Cr_1/4_NbSe_2_ single crystals were synthesized using stoichiometric amounts of niobium, selenium and chromium powders (> 99.99%, Chem PUR), mixed in a molar ratio of 1:2:0.3, following the same procedure. Large shiny black single crystals were found at the lower temperature end of the silica ampoule.

### 8.2 Identification and Characterization

#### 8.2.1 Single Crystal X‐Ray Diffraction (Sc‐XRD)

Sc‐XRD was carried out using a BRUKERVENTURE8 with the APEX3 software for the data collection and integration. For the crystal structure solution Jana2006 [54] was used together with SUPERFLIP [55].

#### 8.2.2 Light Microscopy

A high‐resolution digital microscope (Keyence Deutschland GmbH VHX‐7000) was used to take images of the grown bulk crystals after the growth.

#### 8.2.3 Scanning Electron Microscopy (SEM) and Energy‐Dispersive X‐Ray (EDX) Analysis

The morphology and the composition of selected bulk crystals were investigated further by using a scanning electron microscope ‘FEI Nova NanoSEM 200’ (10–15 kV acceleration voltage), equipped with an energy dispersive x‐ray spectroscopy (QUANTA 200/400 AMATEX). At least five measurements of different crystals were averaged to calculate the compositions of as‐grown bulk crystals.

#### 8.2.4 Inductively Coupled Plasma Optical Emission Spectrometry (ICP‐OES)

ICP‐OES was performed with an “ARCOS MV” (Spectro). To this aim, ∼ 30 mg of sample material were dissolved and diluted to 10 g H_2_O (l) per 1 mg sample, filtered and this solution used for the quantification. Respective element standards in appropriate concentration were used for the calibration. Each solution was measured three times and averaged, while for each material sample again three separate measurement cycles were performed and averaged for the final result. The Cr_x_NbSe_2_ samples were dissolved by mixing the material with 2 mL HNO_3_ (p.a. 65%) and 0.5 mL HF (p.a. 40%), heating to 80°C until a complete dissolution of the material occurred.

#### 8.2.5 High‐Resolution Transmission Electron Microscopy (HR‐TEM)

High resolution transmission electron microscopy (HR‐TEM) was conducted at an aberration‐ (Cs) corrected TF Titan 80–300 (ThermoFisher Scientific) instrument operated at an acceleration voltage of 300 kV. The software package “SingleCrystal Version 5.2” (CrystalMaker software Ltd.) was employed to compute electron diffraction patterns from the corresponding structure mode. TEM lamella were prepared by focused ion beam (FIB) milling for TEM measurements for cross‐sectional high‐resolution imaging of the both as‐grown pristine NbSe_2_ and intercalated Cr_1/4_NbSe_2_ bulk crystals, employing a Helios Nanolab 600 and a Helios Nanolab 5CX system (Thermo Fisher Scientific). Prior to FIB cutting, a C/Pt protective coating layer was deposited to protect the nanostructures on the substrate. Lamellae were extracted using a standard procedure with a maximum energy of 30 kV and concluding with low voltage polishing steps down to 2 kV after lift‐out.

#### 8.2.6 Raman Spectroscopy

Micro‐Raman measurements were performed at room temperature in back‐scattering configuration using “T64000 spectrometer” (Horiba Jobin Yvon, USA), equipped with liquid nitrogen‐cooled Symphony CCD detector and laser excitations at 532 and 660 nm. To avoid uncontemplated polarization effects, polarization of excitation lasers was scrambled by a depolarizer. For measurements with circular‐polarization, λ/4 plate was mounted right before objective, which transforms linear‐polarized light to circular‐polarized and back.

#### 8.2.7 DFT Calculations of the Phonons

The structure of Cr_1/4_NbSe_2_ was optimized with Vienna ab initio simulation package (VASP) (version 5) using the Perdew–Burke–Ernzerhof functional and the projector augmented wave method (PBE/PAW) [42, 56]. Atomic positions were relaxed while the unit‐cell parameters were kept fixed at the experimental values, achieving a free‐energy convergence better than 10^−6^ eV. The self‐consistent filed (SCF) calculation was performed spin‐polarized with a Γ‐centered 6 × 6 × 2 k‐point mesh. The optimized geometries were then used to build a 2 × 2 × 2 supercells. For each system, within the same PBE/PAW level of theory, a density functional perturbation theory (DFPT) calculation was carried out on this supercell, employing a reduced electronic *k*‐mesh of 2 × 2 × 2 to obtain the Γ‐point dynamical matrix (Hessian). The dynamical matrix was analyzed with Phonopy (version 2.45) [57]. Visualization of vibrational displacements was done with the program V_Sim.

#### 8.2.8 Reflectivity Measurements

The optical reflectivity in the *ab* plane of NbSe_2_ and intercalated Cr_1/4_NbSe_2_ has been determined at room temperature in an energy range of 50 meV to 1.4 eV using a Bruker Vertex spectrometer equipped with a microscope. For these measurements, as grown crystals have been placed under the microscope and oriented to achieve the optimal reflection geometry. A gold mirror has been used as reference.

#### 8.2.9 Transport Measurements

DC electrical transport measurements, including resistivity and magnetoresistance (MR), were performed using a standard four‐probe configuration. Sample for electrical measurements were prepared by attaching electrodes (50‐µm silver wires) with conductive silver paint (Dupont 4929N). Resistivity measurements were carried out from 1.8 K up to room temperature using a Physical Property Measurement System (PPMS) equipped with a 9 T magnet. For out‐of‐plane measurements, the magnetic field was applied along the crystal *c*‐axis, while the electric current flowed within the *ab*‐plane. Here, we define the MR =  100 × {[ρ(*B*) − ρ(0)] / ρ(0)} in which ρ(*B*) and ρ(0) represent the resistivity with and without magnetic field, respectively.

#### 8.2.10 Bulk Magnetometry

Bulk magnetometry measurements to study the magnetization as a function of temperature and applied magnetic field, were performed using a Quantum Design Magnetic Property Measurement System (MPMS 3) using a vibrating sample magnetometer (VSM). Additionally, angular dependent measurements were performed using the QD rotator option for the MPMS 3. AC susceptibility χ‘ as a function of temperature was measured at several excitation frequencies using the AC option of a QD SQUID magnetometer (MPMS‐XL).

#### 8.2.11 Specific Heat

The specific heat of the sample was measured using the relaxation method with a QD Physical Property Measurement System (PPMS). The background of the sample platform and Apiezon grease for good thermal coupling was measured separately and subtracted. The data was further analyzed using the sloped method of the heating and cooling regions of the relaxation measurement.

#### 8.2.12 Electron Spin Resonance (ESR)

ESR experiments were performed using a homemade high‐field/high‐frequency (HF‐ESR) spectrometer. A superconducting magnet with a variable temperature inserts (VTI, Oxford Instruments) was used to generate magnetic field up to 16 T. The sample was mounted on a probe head and inserted into the VTI, enabling temperature dependent measurements in the range of 3–300 K. The microwaves were generated and detected using a vector network analyzer (PNA‐X, Keysight Technologies), equipped with frequency extensions (Virginia Diodes Inc.) enabling an operation in the frequency range of 75 to 330 GHz. The measurements were performed in the transmission mode, where the microwaves were delivered to the sample using oversized waveguides. In this configuration, Cr_1/4_NbSe_2_ was measured in two orientations of magnetic field with respect to the sample: in‐plane (*H* ∥ *ab*) and out‐of‐plane (*H* ⊥ *ab*). All measurements were performed by continuously varying the magnetic fields from 0 to 16 T and back at a constant temperature and frequency. Note, that the shape of the presented here ESR spectra was corrected to eliminate the dispersion component of the detected signal as explained in reference [58].

## Author Contributions

N.A., S.H and B.B. conceived and designed the project. N.A. performed material synthesis and analysis, optical microscopy, SEM/EDX characterization and analysis, as well as single‐crystal XRD analysis. M.V. carried out single‐crystal‐XRD measurements, D.W. conducted HR‐TEM measurements. A.P contributed to the Raman spectroscopy analysis. S.M.A. performed DFT‐calculations of the phonons. M.K. and M.N. implemented reflectivity measurements and analysis, D.E. performed DFT calculations, M.B. carried out resistivity and magneto‐transport measurements and their analysis. B.R. and A.W. conducted bulk magnetometry and specific heat measurements with their analysis, M.F.D.B., V. K. and A.A. performed ESR measurements and analysis. The manuscript was drafted by N.A., with input from all authors. S.H., B.B. and M.M. supervised the project.

## Conflicts of Interest

The authors declare no conflicts of interest.

## Supporting information




**Supporting File**: advs78151‐sup‐0001‐SuppMat.docx.

## Data Availability

The data that supports the findings of this study are available in the supplementary material of this article.
