# Supplementary material for: Cr1/4NbSe2‐Single Crystal Growth and Evolution of Antiferromagnetic Correlated States
Source: Adv Sci (Weinh). 2026 Oct 4:e78151. Online ahead of print. doi: 10.1002/advs.78151 (PMC13634576; doi:10.1002/advs.78151)
Supplement: Supplementary file 1 [file ADVS-9999-e78151-s001.docx]

Supporting Information for

**Cr_1/4_NbSe_2_ - Single Crystal Growth and Evolution of Antiferromagnetic Correlated States**

*Nour Abdelrahman***, Bastian Rubrecht*, [*Matheus Felipe de Souza Barbosa*](https://www.researchgate.net/scientific-contributions/Matheus-Felipe-de-Souza-Barbosa-2269376597?_tp=eyJjb250ZXh0Ijp7ImZpcnN0UGFnZSI6InNjaWVudGlmaWNDb250cmlidXRpb25zIiwicGFnZSI6InB1YmxpY2F0aW9uIiwicHJldmlvdXNQYWdlIjoic2NpZW50aWZpY0NvbnRyaWJ1dGlvbnMifX0)*, Mahdi Behnami, Dmitriy Efremov, Daniel Wolf, Martin Valldor, Alexey* *A. Popov,* *Stanislav M. Avdoshenko,* *Marco Naumann, Martin Knupfer,* *Vladislav Kataev, Alexey Alfonsov, Anja U.B. Wolter,* *Michael Mertig, Bernd Büchner, and Silke Hampel*

**Cr_1/4_NbSe_2_ - Single Crystal Growth and and Evolution of Antiferromagnetic Correlated States**

*Nour Abdelrahman^abd^*, Bastian Rubrecht^a^*, [*Matheus Felipe de Souza Barbosa*](https://www.researchgate.net/scientific-contributions/Matheus-Felipe-de-Souza-Barbosa-2269376597?_tp=eyJjb250ZXh0Ijp7ImZpcnN0UGFnZSI6InNjaWVudGlmaWNDb250cmlidXRpb25zIiwicGFnZSI6InB1YmxpY2F0aW9uIiwicHJldmlvdXNQYWdlIjoic2NpZW50aWZpY0NvbnRyaWJ1dGlvbnMifX0)*^a^, Mahdi Behnami^a^, Dmitriy Efremov^a^, Daniel Wolf^a^, Martin Valldor^d^, Alexey A. Popov^a^,* *Stanislav M. Avdoshenko^a^,* *Marco Naumann^a^, Martin Knupfer^a^,* *Vladislav Kataev^a^, Alexey Alfonsov^a^, Anja U.B. Wolter^a^, Michael Mertig^be^, Bernd Büchner^afg^, and Silke Hampel^a^*

a. Leibniz Institute for Solid State and Materials Research Dresden, Helmholtzstraße 20, 01069 Dresden, *Germany*.

b. Institute of Physical Chemistry, Technische Universität Dresden, 01062 Dresden, Germany

c. Helmholtz-Zentrum Dresden-Rossendorf e.V.01328 Dresden, *Germany*.

d. Department of Chemistry, University of Oslo, NO-0315 Oslo, *Norway*.

e. Kurt-Schwabe-Institut für Mess- und Sensortechnik Meinsberg e.V., Kurt-Schwabe-Straße 4, 04736 Waldheim, Germany.

f. Institute of Solid State and Materials Physics. Technische Universität Dresden, 01062 Dresden, *Germany*.

g. Würzburg-Dresden Cluster of Excellence ctd.qmat. Technische Universität Dresden, 01062 Dresden, *Germany*.

I. **Synthesis, Characterization and Crystal Structure**

**Figure S1.** Optical microscopy images of as-grown Cr_1/4_NbSe_2_ single crystals obtained by CVT, revealing the high-yield and the demonstrating screw-dislocation growth mode with a hexagonal morphology.

**Figure S2.** EDX spectrum (15 kV) of Cr_1/4_NbSe_2_ single crystals. (a) After mechanical exfoliation. (b) Before mechanical exfoliation.

**Table S1.** ICP-OS results and calculated composition of the synthesized Cr_1/4_NbSe_2_ single crystals.

| Sample name | | Elements to be analyzed | | | Elemental ratio |
| --- | --- | --- | --- | --- | --- |
| Single crystal Cr_1/4_NbSe_2_ | Cr | | Nb | Se | Cr : Nb : Se |
|  | 4.39 | | 35.34 | 59.57 | 0.22: 1.00: 1.98 |

**Table S2**. Summary of crystallographic data for Cr_1/4_NbSe_2_ from scXRD.

| Empirical formula | Cr_1/4_NbSe_2_ |
| --- | --- |
| Formula weight (g mol^−1^) | 263.8 |
| Temperature (K) | 293 |
| Crystal system | hexagonal |
| Space group | *P*63/*mmc* |
| a (Å) | 6.9062(2) |
| c (Å) | 12.5795(4) |
| a, β, γ (°) | 90, 90, 120 |
| Volume (Å^3^) | 519.60(3) |
| Z | 8 |
| rcalc (g cm^−3^) | 6.745 |
| μ (mm^−1^) | 33.25 |
| F(000) | 920 |
| Radiation | Mo Kα (λ = 0.71073) |
| 2Ө range (°) | 6.48 to 102.92 |
| Index ranges | −7 ≤ h ≤ 0, 0 ≤ k ≤ 15, 0 ≤ l ≤ 27 |
| Reflections collected | 49293 |
| Independent reflections | 1146 [Rint = 0.0733, Rsigma = 0.0203] |
| Data / restraints / parameters | 1146 / 0 / 18 |
| Goodness-of-fit on all F^2^ | 1.91 |
| Final R indexes [I>=3σ (I)] | R = 0.0567, wR2 = 0.1594 |
| Final R indexes [all data] | R = 0.0708, wR2 = 0.1769 |
| Largest diff. peak/hole (e Å^-3^) | 3.22 / −12.18 |

**Table S3.** Comparative summary of crystallographic data highlighting structural changes induced by Cr intercalation in 2*H*‑NbSe₂.

|  | 2*H*-NbSe_2_ 16304-ICSD | Cr_1/4_NbSe_2_ |
| --- | --- | --- |
| Crystal system | hexagonal | hexagonal |
| Space group | *P*6_3_/*mmc* | *P*6_3_/*mmc* |
| *a*/Å | 3.4446 | 6.9062(2) |
| *b*/Å | 3.4446 | 6.9062(2) |
| c/Å | 12.5444 | 12.5795 |
| *α*/° | 90 | 90 |
| *β*/° | 90 | 90 |
| *γ*/° | 120 | 120 |
| Volume/Å^3^ | 128.9016 | 519.60(3) |

**Figure S3.** Side view of the Cr_1/4_NbSe_2_ crystal structure, showing the different positions of the atoms.

**Table S4.** Measured atomic distances, obtained using DIAMOND software.

| Atom 1 | Atom 2 | Distance (Å) |
| --- | --- | --- |
| Cr1 | Cr1 | 6.2898 |
| Cr1 | Nb1 | 4.6715 |
| Cr1 | Nb2 | 3.1449 |
| Cr1 | Se1 | 4.2296 |
| Cr1 | Se2 | 2.5084 |

**Table S5**. Wyckoff positions of the atoms in Cr_1/4_NbSe_2_, obtained using DIAMOND software_._

| Atom, Wyckoff | *x*,*y*,*z*,*U*_iso_,*U*_11_,*U*_22_,*U*_33_,*U*_12_,*U*_13_,*U*_23_ |
| --- | --- |
| Nb1, 6*h* | 0.49208(4), 0.50792(4), 3/4, 0.0036(1), 0.0039(1), 0.0039(1), 0.0027(2), 0.0018(1), 0, 0 |
| Nb2, 2*b* | 0, 0, 3/4, 0.0041(1), 0.0041(2), 0.0041(2), 0.0040(2), 0.00206(9), 0, 0 |
| Se1, 4*f* | 1/3, 2/3, 0.61216(5), 0.0049(1), 0.0057(2), 0.0057(2), 0.0032(2), 0.00287(9), 0, 0 |
| Se2, 12*k* | 0.83145(3), 0.66289(6), 0.61863(3), 0.0046(1), 0.0051(2), 0.0049(2), 0.0036(2), 0.00246(8), 0.00039(4) 0.00078(7) |
| Cr1, 2*a* | 0, 0, 0.5, 0.0057(2), 0.0068(3) 0.0068(3) 0.0034(4), 0.0034(2), 0, 0 |

II. **Raman Study**

**Table S6.** Calculated and experimental frequencies (cm^−1^) of *A*_1_*_g_* and *E*_2_*_g_* modes in Cr_1/4_NbSe_2_ and comparison to Fe_1/4_NbSe_2_. Data on Fe_1/4_NbSe_2_ are extracted from reference. ^[1]^

| Sym. | Calc. Cr | Exp. Cr | Exp. Fe | Sym. | Calc. Cr | Exp. Cr | Exp. Fe |
| --- | --- | --- | --- | --- | --- | --- | --- |
| *A*_1_*_g_*^1^ | 151.2 | 139 vs | 125 | *E*_2_*_g_*^1^ | 51.5 |  |  |
| *A*_1_*_g_*^2^ | 218.5 | 207 m | ~205 | *E*_2_*_g_*^2^ | 97.1 | 100 s | 97 |
| *A*_1_*_g_*^3^ | 246.4 | 232 m | 226 | *E*_2_*_g_*^3^ | 131.7 | 139 w (?) | 125 (?) |
| *A*_1_*_g_*^4^ | 277.5 | 270 s | 263 | *E*_2_*_g_*^4^ | 205.8 |  |  |
|  |  |  |  | *E*_2_*_g_*^5^ | 225.8 |  |  |
|  |  |  |  | *E*_2_*_g_*^6^ | 250.1 | 256 m | 248 |
|  |  |  |  | *E*_2_*_g_*^7^ | 264.8 | 260 m | 253 |

Standard spectroscopic intensity designations: s = strong, m = medium, w = weak, vs = very strong, (?) = ambiguous assignment either due to very low intensity, signals overlap of signals or additional unidentified causes reason.

Vibrational representation of the Cr_1/4_NbSe_2_ crystal in Γ-point spans the following irreducible representations of the *D*_6_*_h_* factor group:

Γ_vib_ = 4*A*_1_*_g_*(R) + 2*A*_2_*_g_* + 5*B*_1_*_g_* + *B*_2_*_g_* + 5*E*_1_*_g_*(R) + 7*E*_2_*_g_*(R) + *A*_1_*_u_* + 6*A*_2_*_u_*(IR) + 2*B*_1_*_u_* +5*B*_2_*_u_* +8*E*_1_*_u_*(IR) + 6*E*_2_*_u_*, where R and IR denotes Raman and IR activity.

**Table S7.** DFT-computed Γ-point vibrational modes of Cr_1/4_NbSe_2_ (frequencies in cm^−1^).

| Sym. | Freq. | Sym. | Freq. | Sym. | Freq. | Sym. | Freq. |
| --- | --- | --- | --- | --- | --- | --- | --- |
| *A*_1_*_g_*^1^ | 151.2 | *E*_1_*_g_*^1^ | 121.0 | *A*_1_*_u_* | 166.0 | *E*_1_*_u_*^1^ | 0.0 |
| *A*_1_*_g_*^2^ | 218.5 | *E*_1_*_g_*^2^ | 148.3 |  |  | *E*_1_*_u_*^2^ | 77.4 |
| *A*_1_*_g_*^3^ | 246.4 | *E*_1_*_g_*^3^ | 176.1 | *A*_2_*_u_*^1^ | 0.0 | *E*_1_*_u_*^3^ | 115.4 |
| *A*_1_*_g_*^4^ | 277.5 | *E*_1_*_g_*^4^ | 191.3 | *A*_2_*_u_*^2^ | 137.8 | *E*_1_*_u_*^4^ | 200.1 |
|  |  | *E*_1_*_g_*^5^ | 252.8 | *A*_2_*_u_*^3^ | 183.1 | *E*_1_*_u_*^5^ | 213.1 |
| *A*_2_*_g_*^1^ | 101.5 |  |  | *A*_2_*_u_*^4^ | 245.8 | *E*_1_*_u_*^6^ | 231.7 |
| *A*_2_*_g_*^2^ | 226.6 | *E*_2_*_g_*^1^ | 51.5 | *A*_2_*_u_*^5^ | 269.7 | *E*_1_*_u_*^7^ | 256.4 |
|  |  | *E*_2_*_g_*^2^ | 97.1 | *A*_2_*_u_*^6^ | 300.5 | *E*_1_*_u_*^8^ | 264.5 |
| *B*_1_*_g_*^1^ | 70.5 | *E*_2_*_g_*^3^ | 131.7 |  |  |  |  |
| *B*_1_*_g_*^2^ | 137.9 | *E*_2_*_g_*^4^ | 205.8 | *B*_1_*_u_*^1^ | 116.6 | *E*_2_*_u_*^1^ | 125.5 |
| *B*_1_*_g_*^3^ | 208.6 | *E*_2_*_g_*^5^ | 225.8 | *B*_1_*_u_*^2^ | 220.8 | *E*_2_*_u_*^2^ | 134.8 |
| *B*_1_*_g_*^4^ | 277.5 | *E*_2_*_g_*^6^ | 250.1 |  |  | *E*_2_*_u_*^3^ | 167.1 |
| *B*_1_*_g_*^5^ | 301.6 | *E*_2_*_g_*^7^ | 264.8 | *B*_2_*_u_*^1^ | 101.9 | *E*_2_*_u_*^4^ | 171.1 |
|  |  |  |  | *B*_2_*_u_*^2^ | 140.0 | *E*_2_*_u_*^5^ | 234.5 |
| *B*_2_*_g_* | 151.4 |  |  | *B*_2_*_u_*^3^ | 200.6 | *E*_2_*_u_*^6^ | 247.4 |
|  |  |  |  | *B*_2_*_u_*^4^ | 255.2 |  |  |
|  |  |  |  | *B*_2_*_u_*^5^ | 258.4 |  |  |

 **Figure S4.** Vibrational displacements of *A*_1g_ modes of Cr_1/4_NbSe_2_.

**Figure S5.** Vibrational displacements of *E*_2g_ modes of Cr_1/4_NbSe_2_.

III. **Density-Functional-Theory (DFT) Calculations**

Figure S6 illustrates the effect of spin-orbit coupling (SOC) on the electronic band structure. It compares the electron band structure in the spin-nonpolarized state, calculated within the full-potential local-orbital band structure (FPLO) using the full-relativistic functional (with SOC) and the scalar-relativistic functional (without SOC). SOC primarily lifts degeneracies and induces additional band splitting near the M and K points, while leaving the overall band dispersion largely unchanged.

**Figure S6.** Non-spin-polarized electronic band structures calculated using the full-relativistic approach (with SOC) and the scalar-relativistic approach (without SOC) for Cr_1/4_NbSe_2_ (a) and NbSe_2_ (b).

Figure S7 presents the orbital- and spin-resolved density of states (DOS) of a single Cr atom in Cr_1/4_NbSe_2_, calculated for three magnetic configurations: the ferromagnetic state (FM), a state with ferromagnetic planes coupled antiferromagnetically (A-AFM), and a stripe-ordered in-plane state with ferromagnetic interplane coupling (C-AFM). The corresponding band structure for illustration is shown in Figure S8. In all cases, the Cr 3d states are distributed over a broad energy range extending from approximately − 6 to 5 eV. The spectra are strongly spin polarized, with a pronounced exchange splitting between the two spin channels. Although the relative weight of the individual 3d orbitals varies somewhat between the three magnetic states, the overall electronic structure of the Cr-derived states remains qualitatively similar. Integrating the spin-resolved DOS yields a local moment corresponding to an effective spin of approximately S = 3/2 in all three cases, indicating that the Cr moment is robust with respect to the magnetic arrangement.

**Figure S7.** Spin-resolved projected density of states (PDOS) of the Cr 3d orbitals for: a) ferromagnetic state, b) ferromagnetic planes with antiferromagnetic interplane coupling, c) striped-ordered in-plane with ferromagnetic interplane coupling. Positive and negative values correspond to opposite spin channels.

**Figure S8**. Spin- and orbital-resolved band structure in the FM state. The coloured markers denote the spin-resolved orbital contributions to the bands, with marker size proportional to the weight of the corresponding Cr 3𝑑 orbital. Solid and dashed lines indicate the two spin channels.

A population analysis within FPLO shows that the 3d electrons of Cr make the dominant contribution to magnetism. Figure S9 illustrates the orbital contribution of 3d orbitals to the low-energy electronic structure in the two magnetic configurations: ferromagnetic and A-type antiferromagnetic. Overall, the comparison reveals that magnetic order primarily influences spin splitting and the energetic position of the Cr-derived bands, while the dominant orbital character around the Fermi level remains largely unchanged.

**Figure S9**. The orbital-resolved electronic band structures of the Cr 3d states are shown for the two considered magnetic configurations: (a) ferromagnetic (FM) and (b) A-type AFM. Colored markers indicate the orbital character of the bands, with their size proportional to the weight of the corresponding Cr 3d orbital. The solid and dashed lines represent the two spin channels.

IV. **Resistivity and Magneto-transport Measurements**

The electrical resistivity of pristine 2*H*–NbSe₂ and Cr_1/4_NbSe₂ single crystals was systematically investigated and compared. Figure S7a shows the temperature-dependence resistivity of 2*H*-NbSe_2_ under constant magnetic fields ranging from *B* = 0 T to 9 T. At zero field, the resistivity exhibits a sharp drop to zero near 6.8 K, corresponding to the superconducting transition temperature (*T*_c_), as indicated by the dark blue curve in Figure S 10a. With increasing magnetic field, the transition shifts to lower temperatures, demonstrating that higher fields progressively suppress superconductivity, requiring lower temperatures to sustain it. This behavior is characteristic of type II superconductor, ^[2-4]^ where magnetic fields break Cooper pairs, reducing *T*_c_. At *B* = 9 T, the superconducting transition disappears entirely, defining the upper critical field (*B*_C2_), illustrated by the brown curve in Figure S 10a. Figure S 10b presents the magneto-resistivity as a function of magnetic field at *T* = 2 K and 5 K. At *T* = 2 K, the system is deep within the superconducting state, thus, the resistivity remains nearly zero at low fields. Once, the applied field exceeds *B*_C2_, the resistance rises sharply to its normal-state value, producing a pronounced “V-sharp” curve that reflects the abrupt loss of superconductivity beyond the critical field. At 5 K, closer to *T*_c_, superconductivity is already weakened, resulting in a narrower zero-resistance region, because B_(C2)_ is smaller at this temperature

**Figure S10.** (a) Temperature-dependent resistivity of 2H-NbSe_2_ under various constant magnetic fields. (b) Magnetoresistance of 2H-NbSe_2_ as a function of applied magnetic field at different temperatures.

V. **AC susceptibility Measurements**

**Figure S11.** The real contribution of AC magnetic susceptibility χ‘ as a function of temperature measured at various frequencies with zero applied DC field and an AC amplitude of 2.5 Oe.

**REFERENCES**

[1] Erodici, M. P.; Mai, T. T.; Xie, L. S.; Li, S.; Fender, S. S.; Husremović, S.; et al. Bridging Structure, Magnetism, and Disorder in Iron-Intercalated Niobium Diselenide, FeₓNbSe₂, below x = 0.25. *J. Phys. Chem. C* 2023, 127, 9787–9795.

[2] Foner, S.; McNiff, E. J., Jr. Upper Critical Fields of Layered Superconducting NbSe₂ at Low Temperature*. Phys. Lett. A* 1973, 45, 429–430.

[3] Xi, X.; Wang, Z.; Zhao, W.; Park, J.-H.; Law, K. T.; Berger, H.; Forró, L.; Shan, J.; Mak, K. F. Ising Pairing in Superconducting NbSe₂ Atomic Layers. *Nat. Phys*. 2016, 12, 139–143.

[4] S.; Li, Y.; Liu, Y.; Li, Z.; Qin, L.; Wu, H.; Xing, Y. Growth of 2D van der Waals Superconductor–Ferromagnet NbSe₂–VSe₂ Heterostructure by CVD Method. *Mater. Lett.* 2025, 138603.
